# Imputing single-cell RNA-seq data by considering cell heterogeneity and prior expression of dropouts

**DOI:** 10.1093/jmcb/mjaa052

**Published:** 2020-10-01

**Authors:** Lihua Zhang, Shihua Zhang

**Affiliations:** 1 NCMIS, CEMS, RCSDS, Academy of Mathematics and Systems Science, Chinese Academy of Sciences, Beijing 100190, China; 2 School of Mathematical Sciences, University of Chinese Academy of Sciences, Beijing 100049, China; 3 Center for Excellence in Animal Evolution and Genetics, Chinese Academy of Sciences, Kunming 650223, China; 4 Key Laboratory of Systems Biology, Hangzhou Institute for Advanced Study, University of Chinese Academy of Sciences, Chinese Academy of Sciences, Hangzhou 310024, China

**Keywords:** single-cell RNA-seq, dropout, imputation, low-rank, systems biology

## Abstract

Single-cell RNA sequencing (scRNA-seq) provides a powerful tool to determine expression patterns of thousands of individual cells. However, the analysis of scRNA-seq data remains a computational challenge due to the high technical noise such as the presence of dropout events that lead to a large proportion of zeros for expressed genes. Taking into account the cell heterogeneity and the relationship between dropout rate and expected expression level, we present a cell sub-population based bounded low-rank (PBLR) method to impute the dropouts of scRNA-seq data. Through application to both simulated and real scRNA-seq datasets, PBLR is shown to be effective in recovering dropout events, and it can dramatically improve the low-dimensional representation and the recovery of gene‒gene relationships masked by dropout events compared to several state-of-the-art methods. Moreover, PBLR also detects accurate and robust cell sub-populations automatically, shedding light on its flexibility and generality for scRNA-seq data analysis.

## Introduction

Single-cell RNA sequencing (scRNA-seq) has made a grand advance on throughput and resolution, providing a promising tool to study heterogeneous systems (Nawy, 2014). However, the quantity of mRNA in a single cell is so tiny that a million-fold amplification is often used. Therefore, only a fraction of transcripts may be captured during library preparation and a large amplification noise may be introduced during this stage. The low RNA capture rate might lead to failure of detecting an expressed gene, resulting in a false zero count observation, which is called ‘dropout’ event (Kharchenko et al., 2014). Thus, pervasive ‘dropout’ events exist in scRNA-seq data, in which genes have false zero or near zero expression in some cells.

High ratio of ‘dropout’ may mislead downstream analyses such as low-dimensional representation, cell sub-population identification and cellular trajectory reconstruction. Several imputation methods have been developed to address such potential issue in scRNA-seq data (Zhang and Zhang, 2020). These imputation methods have various model assumptions, and usually model the missing value of a given gene in a specific cell according to the expression level of its co-expressed genes. For example, MAGIC (van Dijk et al., 2018) reconstructs the gene expression profile by a Markov affinity graph. scImpute (Li and Li, 2018) divides values into ‘dropout’ ones that need to be imputed and ‘confident’ ones that are not affected by dropout events with a mixture model, and then imputes ‘dropout values’ with a non-negative least square model in an individual cell. SAVER (Huang et al., 2018) and BISCUIT (Prabhakaran et al., 2016) are two Bayesian-based methods. Our comprehensive comparison analyses (Zhang and Zhang, 2020) indicate that scImpute may perform not very good on data with less collinearity, and SAVER and BISCUIT often impute dropouts with near zero values. Most recently, several other imputation methods have been proposed, such as deep learning-based method DCA (Eraslan et al., 2019) and deepImpute (Arisdakessian et al., 2019), matrix factorization-based method CMFImpute (Xu et al., 2020), and low-rank-based method ALRA (Linderman et al., 2018). Low-rank matrix recovery method that approximates a low-rank matrix based on a few observable entries is a direct and powerful imputation strategy, which has shown promising performance in many fields (Candes and Recht, 2009). A recent study suggests that taking advantages of the presence of low-rank submatrices improves the performance compared to the traditional low-rank recovery (Ruchansky et al., 2017). scRNA-seq data exhibit high heterogeneity, implying the existence of structured low-rank submatrices. Moreover, a previous study showed that gene expression levels have distinct effects on the dropout events (Kharchenko et al., 2014). Existing imputation methods rarely take into account this particular structural feature of single-cell expression data. Thus, integrating these characteristics into one framework to achieve an effective recovery of gene expression level is of great potential.

To this end, we present a novel cell sub-population based bounded low-rank (PBLR) method for scRNA-seq data imputation, which considers the cell heterogeneity and the effects of gene expression on dropouts. Applications to both simulated and real scRNA-seq data suggest that PBLR is an effective tool to recover transcriptomic level and dynamics masked by dropouts, improve low-dimensional representation, and restore the gene‒gene co-expression relationship. Moreover, PBLR is also able to accurately identify cell sub-populations.

## Results

### Overview of PBLR

**Figure 1:**
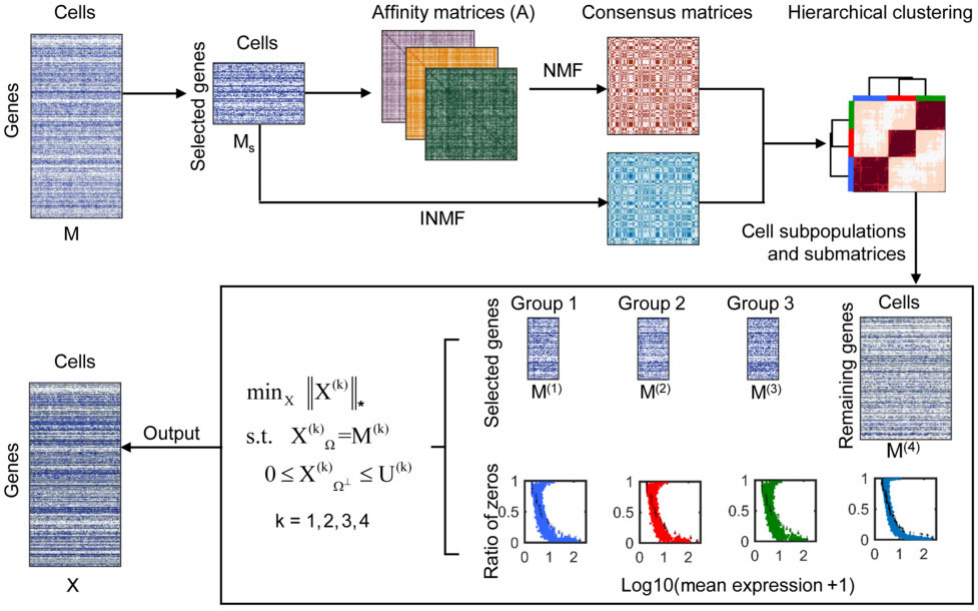
Overview of PBLR. Given a gene expression matrix *M* as input, PBLR outputs an imputed data matrix *X* with the same size as *M*. PBLR first extracts the data of the selected high variable genes and computes three affinity matrices based on Pearson, Spearman, and Cosine metrics, respectively. Then, PBLR learns a consensus matrix by performing SymNMF of the three affinity matrices INMF of the sub-matrix of selected genes. PBLR further infers cell sub-populations by performing hierarchical clustering of the consensus matrix. Finally, PBLR estimates the expression upper boundary of the ‘dropout’ values, and recovers zero gene expressions by performing a bounded low-rank recovery model on each submatrix determined by each cell sub-population. In this diagram, there are three cell sub-populations. *M*^(1)^, *M*^(2)^, *M*^(3)^ are the sub-matrices of the selected genes for each population, and *M*^(4)^ is the sub-matrix of the remaining genes.

PBLR aims to impute zeros using scRNA-seq data *M* with *m* genes and *n* cells, where *M_ij_* is the expression value of gene *i* in cell *j*. PBLR consists of two components: (i) an ensemble clustering of the scRNA-seq data of the informative genes to determine *g* cell sub-populations; (ii) given the g corresponding submatrices (*M*^(^^*k*^^)^, *k *=* *1, …, *g*) and the submatrix constructed by the remaining genes (*M*^(^^*k*^^)^, *k *=* g *+* *1), a bounded low-rank matrix recovery model is performed on each submatrix *M*^(^^*k*^^)^ (Figure 1). Specifically, PBLR first extracts a set of highly variably expressed genes. PBLR then builds a consensus matrix by employing either symmetric non-negative matrix factorization (SymNMF) and incomplete NMF (INMF) on several affinity matrices or Leiden algorithm (Traag et al., 2019) on shared nearest neighbor (SNN) graph with various resolution values. This inferred consensus matrix is further used as the input of hierarchical clustering to determine final cell sub-populations and submatrices (see Materials and methods).

Let *X*^(^^*k*^^)^ represent the imputed data submatrix corresponding to the *k*th submatrix *M*^(^^*k*^^)^. The low-rank recovery problem is formulated as

$$\begin{array}{c} \min_{X^{\left(k\right)}}{\left\|X^{\left(k\right)}\right\|}_{*}\\ s.t.\;X_{\Omega }{}^{\left(k\right)}=M^{\left(k\right)}, \end{array}$$

where Ω represents the so-called observed space in *M*^(^^*k*^^)^ (i.e. the non-zero space), ${\left\|\cdot \right\|}_{*}$denotes the nuclear norm. Moreover, a recent study has shown that the probability of each gene’s dropout events varies across the expression magnitude, and there is a negative correlation relationship between the dropouts’ expression and the ratio of zeros (Kharchenko et al., 2014). Thus, the upper boundary of dropout values for a gene could be estimated in advance based on its observed expression level in other cells, which will likely improve the recovery accuracy. Therefore, by introducing upper boundaries for unobserved variables, the bounded low-rank matrix recovery model is formulated as

$$\begin{array}{c} \min_{X^{\left(k\right)}}{\left\|X^{\left(k\right)}\right\|}_{*}\\ s.t.\;X_{\Omega }{}^{\left(k\right)}=M^{\left(k\right)},0\leq X_{\Omega^{⊥}}^{\left(k\right)}\leq U^{\left(k\right)}, \end{array}$$

where $\Omega^{⊥}$ represents the unobserved space or say zero space, *U*^(^^*k*^^)^ is a matrix in which each row denotes the upper boundary of a gene expression in the *k*th submatrix *M*^(^^*k*^^)^. This model is solved by an efficient alternating direction method of multipliers (ADMM) algorithm (Gabay and Mercier, 1976; Chen et al., 2012). PBLR obtains the final imputed matrix *X* by merging these imputed submatrices *X*^(^^*k*^^)^.

### PBLR recovers dropouts with superior accuracy on eight synthetic datasets

To evaluate the imputation performance of PBLR, we generated eight synthetic datasets by Splatter (Zappia et al., 2017). Each dataset was designed as one of the eight scenarios to account for different data properties, such as fixed or varied dropout rates, discrete or continuous cell states, and single or multiple paths. Specifically, dataset 1 contains three cell sub-populations with different dropout rates in each sub-population; dataset 2 is a set of data with increasing dropout rates; dataset 3 contains three cell sub-populations with the same dropout rates in each sub-population; dataset 4 contains six cell sub-populations with imbalanced proportions of cells; dataset 5 describes a continuous cell trajectory with a single path; dataset 6 describes a continuous cell trajectory with two paths; dataset 7 describes a continuous cell trajectory with multiple paths; and dataset 8 is a set of data with varied cluster distance and degree of noise.

Compared to the typical low-rank discovery model, PBLR considers the structured characteristics of the data and expression distribution reflected by the observed data to account for both cell- and gene-specific features of scRNA-seq data. To demonstrate the superior performance of these two key components, we used synthetic dataset 1 with dropouts (Supplementary Table S1). By visualizing cells in the low-dimensional space and quantifying the reconstructed errors using two measures, i.e. sum of squared error (SSE) and Pearson correlation coefficient (PCC), our results show that PBLR indeed improves imputation accuracy (Supplementary Figure S1).

**Figure 2:**
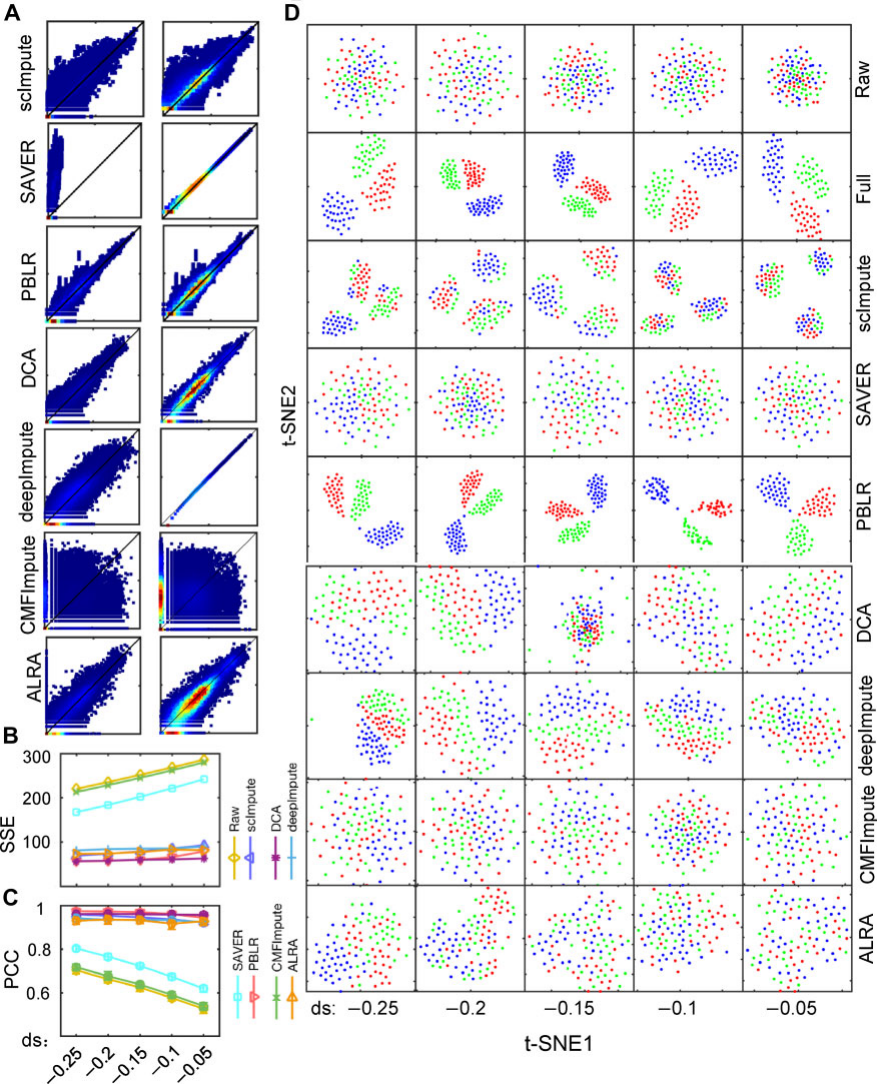
Imputation performance of scImpute, SAVER, PBLR, DCA, deepImpute, CMFImpute, and ALRA on synthetic dataset 2 with various dropout rates. (**A**) Density plot of the imputed values vs. true ones in the zero space (left) and the non-zero space (right), respectively. Y-axis is log10(real value +1), while x-axis is log10(imputed value +1). (**B**) SSE values computed between the full data and the raw data, as well as imputed ones, respectively. (**C**) PCC values computed between the full data and the raw data, as well as imputed ones by scImpute, SAVER, PBLR, DCA, deepImpute, CMFImpute, and ALRA, respectively. (**D**) Visualization of cells on the first two t-SNE components using the raw data and imputed ones by scImpute, SAVER, PBLR, DCA, deepImpute, CMFImpute, and ALRA, respectively. Each column represents data with one dropout rate. ds means the parameter of dropout.shape in splatter package, which controls the ratio of zeros and larger value represents higher ratio of zeros in the data.

To further show the effectiveness of PBLR, we compared it with six competing imputation methods, including scImpute, SAVER, DCA, deepImpute, CMFImpute, and LARA, in terms of the gene expression recovery and the low-dimensional representation. To evaluate performance with respect to different dropout rates, we simulated synthetic dataset 2 with the shape parameters of dropout logistic function (ds) equaling −0.25, −0.20, −0.15, −0.1, −0.05, which correspond to different ratios of zeros varying from 0.6 to 0.71. We divided the entries of raw expression data into zero space and non-zero space. In the zero space, the imputed values of SAVER are much smaller than the real ones, while scImpute gives much larger fluctuations than PBLR (with ds = −0.05 as an example in Figure 2A). These results suggest that PBLR recovers more similar values to the real ones than scImpute and SAVER. In the non-zero space, scImpute treats many moderate expression values as dropouts and imputes them by either larger or smaller values than the real ones (Figure 2A). Moreover, we also evaluated scImpute, SAVER, PBLR, DCA, deepImpute, CMFImpute, and ALRA in terms of the reconstructed errors using SSE and PCC (Figure 2B and C). As expected, the SSE values increase and PCC values decrease with the increase in the ratios of zeros for these imputation methods. All these imputed data improve the performance of SSE and PCC relative to the raw data. Attractively, PBLR and DCA show the smallest SSE values and largest PCC values compared to other imputation methods. Visualization by the first two t-SNE components show that the three cell sub-populations are mixed together due to the existence of large amounts of zeros in raw data. SAVER has hardly any effect on the raw data. scImpute leads to three fictitious cell sub-populations in the t-SNE space, and it shows improved performance in a dataset with a relative larger number of genes (Figure 2D;Supplementary Figure S2A). However, the cell clusters can be well separated after applying PBLR. In summary, PBLR shows a strong ability in recovering dropouts compared to other imputation methods on synthetic dataset 2 with various dropout rates (Figure 2) and synthetic dataset 3 with a relative larger scale (Materials and methods; Supplementary Figure S3). Cell sub-populations were well distinguished after imputation by PBLR and deepImpute on synthetic dataset 4 where the distances between clusters were different (Supplementary Figure S4). Moreover, the underlying cell trajectory was revealed after imputation by SAVER and PBLR on synthetic dataset 5 with a single path (Supplementary Figure S5A). Correlations between the inferred pseudotime after imputation by SAVER and PBLR and real path were higher than other imputation methods (Supplementary Figure S5B and C).

We further evaluated the performance of recovering cell trajectory on dataset 6 with two paths and dataset 7 with multiple paths. To visualize data structure and transitions during differentiation progression, we applied a diffusion-based manifold learning method PHATE (Moon et al., 2019) to the raw data with dropout, full data (i.e. golden standard data without dropout), and imputed data, respectively. By projecting cells into the PHATE space, we found that all methods except for DCA could recover the trajectory structure on synthetic dataset 6 (Supplementary Figure S6A). Notably, PBLR exhibited the most similar trajectory structure with the full data compared to other methods (Supplementary Figure S6A). In addition, we quantified the ability of imputation methods in preserving trajectory structure observed from the low-dimensional space of full data, which was assessed by a manifold preservation score based on the similarity of each cell’s neighborhood in low-dimensional spaces (see Materials and methods). Compared to other methods, scImpute and PBLR had higher preservation scores, suggesting that cells distributed close together in the low-dimensional space of full data show similar trend in the low-dimensional space of imputed data (Supplementary Figure S6B). On dataset 7 with multiple paths, PBLR and deepImpute were able to preserve the global trajectory structure as observed in the PHATE space from the full data. However, the trajectory structure was lost when visualizing the imputed data from other imputation methods (Supplementary Figure S7A). This observation was further confirmed when we computed the manifold preservation metric (Supplementary Figure S7B). Taken together, PBLR consistently show superior performance in recovering dropouts to preserve the trajectory structure and the Euclidean distances among cells in the embedded space.

We used another set of simulation dataset 8 with varied cluster distance and degree of noise to further test the robustness of PBLR. As shown in the Uniform Manifold Approximation and Projection (UMAP) space, PBLR, scImpute, and deepImpute consistently produced similar between-cluster and inter-cluster dispersions as observed in the full data with the increase in cluster distance and degree of noise (Supplementary Figure S8A). However, compared to the full data, SAVER and CMFImpute explicitly decreased between-cluster dispersion, producing less distinguishable clusters in the UMAP space. In addition, we also evaluated the preservation of Euclidean distances between cells by computing the manifold preservation score (see Materials and methods). As expected, PBLR, scImpute, and deepImpute consistently had higher scores than other methods, suggesting the better preservation of cell‒cell distances in the imputed data (Supplementary Figure S8B). Taken together, these results suggest that PBLR has a good control of over-imputation.

### PBLR captures precise expression dynamics during human and mouse embryonic development

**Figure 3:**
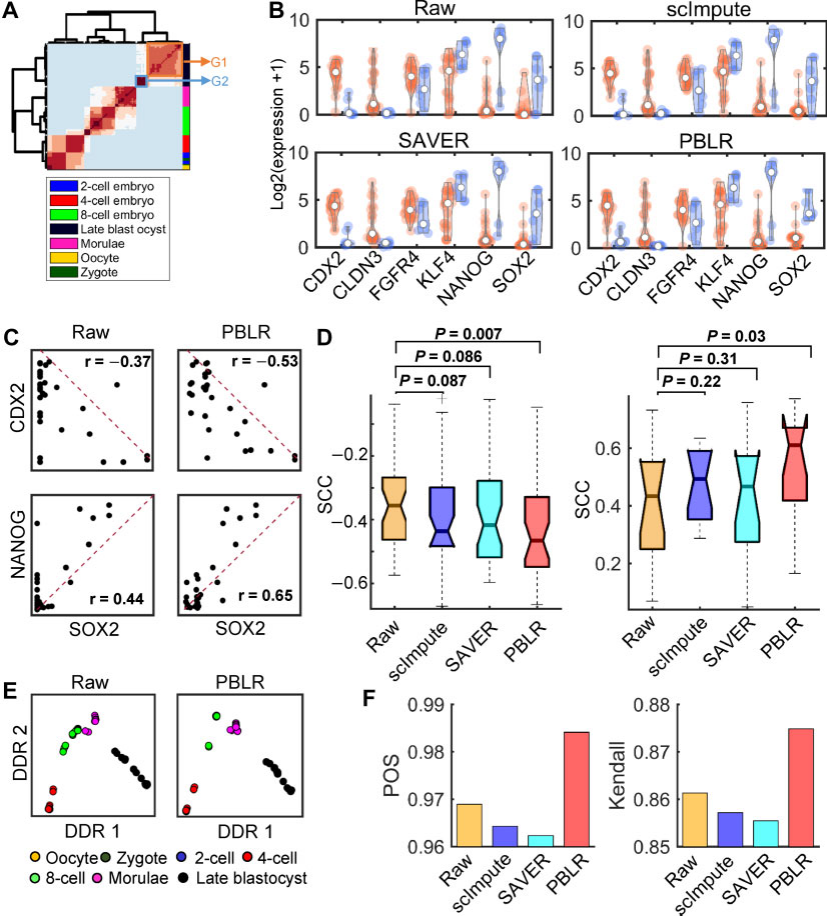
PBLR captures precise expression dynamics of marker genes on the real data from human embryo development. (**A**) Hierarchical clustering of the consensus matrix obtained by PBLR. Experimental stages of individual cells are indicated by different colors on the right. The late blastocyst cells are divided into two groups G1 and G2. (**B**) Violin-plot of gene expression values of marker genes in G1 (orange) and G2 (light blue) groups. (**C**) Scatter plots of the expression levels of marker genes in the raw and imputed data by PBLR, respectively. The corresponding Spearman correlation coefficient (SCC) of expression values in the late blastocyst cells is shown on the top. (**D**) Comparison of SCC values of gene pairs from any two enriched gene sets for TE, EPI, and PE (left) and gene pairs within EPI-specific gene set (right) on imputed data and raw data. Each dot represents a gene pair. *P*-values are computed by one-sided Wilcoxon rank-sum tests. (**E**) Scatter plots of the first two discriminative dimensions inferred by Monocle 2. Each dot represents one cell. (**F**) Bar plots of POS and Kendall’s rank correlation after applying Monocle 2 to the raw and imputed data by scImpute, SAVER, and PBLR, respectively.

First, to show whether the imputation values have biological meaning, we used scRNA-seq data consisting of 88 cells from seven stages (from oocytes to blastocyst) in human early embryos (HEEs) (Yan et al., 2013). Hierarchical clustering of the imputed data with PBLR accurately reveals the similarity of cells in each stage and cells in consecutive stages, and clearly captures the cell sub-populations (Figure 3A). More interestingly, we identified two cell sub-populations (denoted by G1 and G2) at the late blastocyst stage. It has been reported that CDX2 is highly expressed in trophectoderm (TE), SOX2, NANOG, and KLF4 are highly expressed in epiblast (EPI) but lowly expressed in primitive endoderm (PE), and FGFR4 and CLDN3 are highly expressed in PE (Yan et al., 2013). Based on these marker genes, we can see that TE and PE cells are enriched in G1 group, while EPI cells are enriched in G2 group (Figure 3B). Some zero values of these marker genes are imputed by scImpute, SAVER, and PBLR. For example, CDX2 is imputed by scImpute and SAVER. SOX2 is imputed by PBLR (Figure 3B). At the blastocyte stage, two critical segregations take place: the segregations of cells into inner cell mass (ICM) and TE cells, and further differentiation of ICM cells into EPI and PE. Therefore, the expression levels of CDX2 and SOX2 exhibit a negative correlation relationship, while the expression levels of NANOG and SOX2 show positive correlation relationship. After imputation, scImpute, SAVER, and PBLR enhance the relationship of these two pairs of marker genes in different degree (Figure 3C;Supplementary Figure S9A). Attractively, PBLR significantly decreases the correlation between CDX2 and SOX2 from −0.37 to −0.53, and increases the correlation between NANOG and SOX2 from 0.44 to 0.65. To further systematically test the improvement of gene interactions, we downloaded TE, EPI, and PE-enriched marker genes (Supplementary Table S2) from a previous study (Yan et al., 2013). Our results demonstrate that scImpute and SAVER slightly enhance the gene‒gene correlation relationships (*P*-value >0.05, one-sided Wilcoxon rank-sum test), however, PBLR is able to significantly enhance them including both positive and negative correlations (Figure 3D), indicating the effectiveness of PBLR in capturing the subtle expression relationships.

Next, to test whether PBLR can recover gene expression temporal dynamics, we applied Monocle 2 (Qiu et al., 2017) to the imputed data from the HEE development (HEE dataset) and the reprograming from mouse embryonic fibroblasts (MEFs) to induce neuronal (iN) cells (MEF dataset) (Figure 3E;Supplementary Figure S9; Treutlein et al., 2016). The major developmental trajectory can be detected on both raw data and PBLR-imputed data visually inferred by Monocle 2 (Figure 3E;Supplementary Figure S9B). PBLR improves the inference performance distinctly compared to that of raw data and scImpute and SAVER-imputed ones in terms of pseudotime order score (POS) and Kendall’s rank correlation (Figure 3F;Supplementary Figure S9D).

### PBLR exhibits high-quality imputation on large-scale datasets

**Figure 4:**
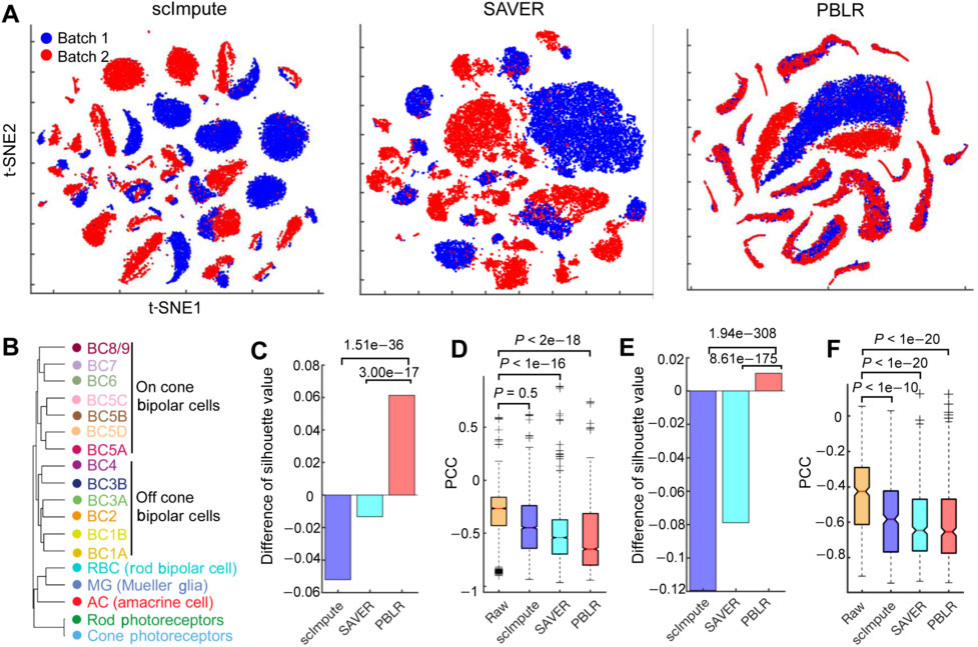
Performance of PBLR on two large-scale datasets. (**A**) Cells (*n *=* *26830) are visualized on the first two t-SNE components using the imputed Shekhar data by scImpute, SAVER, and PBLR. Cells are colored by batches (batch 1: Bipolar 1‒4; batch 2: Bipolar 5 and 6). (**B**) Hierarchical clustering of average gene signatures of clusters based on gene expression imputed by PBLR (Pearson correlation distance metric, average linkage). (**C**) The average difference of silhouette values between each imputed data and raw Shekhar data. *P*-values are computed by performing paired *t*-tests of the distribution of differences of silhouette values from individual cells. (**D**) The PCC values of marker gene pairs from any two different cell types on raw Shekhar dataset and imputed data by scImpute, SAVER, and PBLR, respectively. Each element in boxplot represents a gene pair. *P*-values are computed by one-sided Wilcoxon rank-sum tests. (**E**) The average difference of silhouette values between each imputed data and raw Campbell data. *P*-values are computed by paired *t*-tests. (**F**) The PCC values of marker gene pairs from any two different cell types on raw Campbell data and imputed data by scImpute, SAVER, and PBLR, respectively. Each element in boxplot represents a gene pair. *P*-values are computed by one-sided Wilcoxon rank-sum tests.

We further applied PBLR to two large-scale scRNA-seq datasets to demonstrate its effectiveness and scalability. First, we applied PBLR, scImpute, SAVER, DCA, deepImpute, CMFImpute, and ALRA on a mouse retinal dataset consisting of 26830 cells from two batches (Shekhar et al., 2016). We compared scalability of PBLR with other methods on speed and memory usage (Supplementary Figure S10). PBLR with SNNs strategy performed comparable with DCA, deepImpute, and ALRA in computational speed, while scImpute and SAVER consumed lots of time (for SAVER, we did not obtain the results within one day with 12 cores when the number of cells >10000). scImpute consumed the highest memory. Visually well-separated cell sub-populations in the low-dimensional space are indicative of more meaningful biological conclusions from the data. By projecting cells onto t-SNE using imputed data, PBLR, deepImpute, and CMFImpute provided better mixing of batches in comparison of other methods according to the local inverse Simpson’s index metric (Korsunsky et al., 2019), which measures the local batch distribution based on local neighbors (Figure 4A;Supplementary Figures S11 and S12). In addition, PBLR and CMFImpute well revealed various cell types compared to deepImpute (Supplementary Figure S12).

To further assess the cell sub-population separability, we performed batch effect correction of the raw data and the imputed data using Combat, and again projected cells onto t-SNE space, in which cells were colored by the pre-annotated sub-populations (Supplementary Figure S13A‒C; Shekhar et al., 2016). Rod bipolar cells were still separated into two parts after removing batch effects of the raw data using Combat (Supplementary Figure S13A). More compact and clean cell clusters were shown in the first two t-SNE dimensions using PBLR-imputed data, compared to those from raw data and scImpute and SAVER-imputed data (Supplementary FigureS13B and C). The relatedness of cell clusters can be well revealed by hierarchical clustering of PBLR-imputed data (Figure 4B): the well separation between bipolar cell and non-bipolar cell clusters, cone bipolar cell and rod bipolar cell clusters, as well as on and off cone bipolar cell clusters. However, both raw data and imputed data by other methods cannot well capture the relatedness of BC5A, BC5B, BC5C, and BC5D (Supplementary Figure S13D). In addition, SAVER cannot well separate the on and off cone bipolar cell clusters. To quantitatively assess cell sub-population separability in the 2D space, we used the silhouette index, which is an unsupervised metric to quantify how well each method groups and separates the cells from various sub-populations. To test whether these methods show significant improvement, we computed the difference of silhouette values between each imputed data and raw data. PBLR had better performance than raw data, while scImpute and SAVER were worse than raw data (Figure 4C). scImpute and SAVER borrow information across cells to predict missing expression values, both of them have been shown to improve the gene structure and downstream analyses to some degree on some datasets (Figures 2 and 3). However, the worse low-dimensional representations using scImpute and SAVER than raw data might be due to some unexpected false signals or other biases that were introduced by imputation. Moreover, PBLR can significantly enhance the negative relationships of marker gene pairs from any two different cell clusters, with distinct better performance than those of scImpute and SAVER (one-sided paired *t*-test) (Figure 4D;Supplementary Table S3).

Second, we also computed these quantitative metrics on another dataset, which consists of 20921 cells (including 20 neuron and non-neuron cell types) in and around the adult mouse hypothalamic arcuate-median eminence complex (Campbell et al., 2017). To test the robust ability of PBLR, we randomly downsampled 50% cells. Most cell clusters are separated on both raw and imputed data by PBLR, scImpute, and SAVER (Supplementary Figure S14). Intriguingly, a rare neuron cluster a13 (13 cells) is well separated from the large neuron cluster a18 (10515 cells) after imputed by PBLR and SAVER, while it cannot be distinguished from a18 in scImpute-imputed data. Quantitatively, PBLR had higher silhouette value than raw data, while scImpute and SAVER had smaller silhouette values than raw data (Figure 4E). PBLR, scImpute, and SAVER all significantly enhance the negative relationships of marker gene pairs from any two different cell clusters (Figure 4F;Supplementary Table S4).

### PBLR improves the identification of cell sub-populations on real scRNA-seq datasets

**Figure 5:**
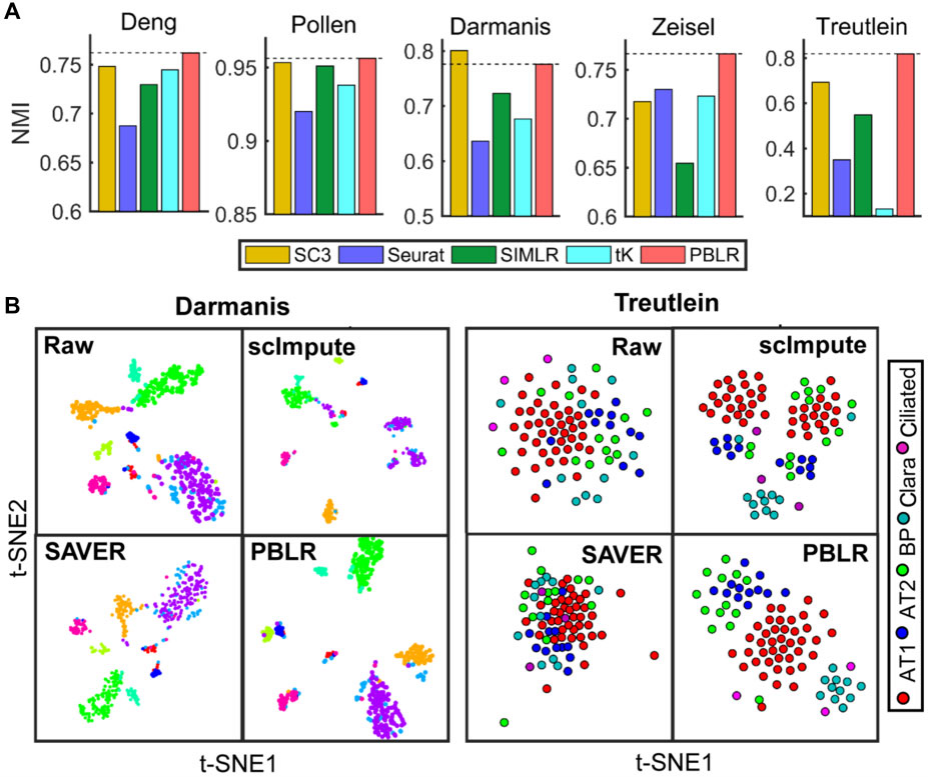
Clustering performance of PBLR and other competing methods on five real datasets. (**A**) SC3, Seurat, SIMLR, tK, and PBLR were applied to the five real scRNA-seq datasets, where cell cluster labels were known or validated in the original studies. tK represents *k*-means on the first two t-SNE dimensions. Normalized mutual information (NMI) is used to quantify accuracy. (**B**) Cells are visualized on the first two t-SNE components using the raw Darmanis (left) and Treutlein (right) data and imputed ones by PBLR, scImpute, and SAVER, respectively.

PBLR can not only impute dropout events, but also reveal cell sub-populations directly from the raw data by an ensemble clustering strategy (see Materials and methods). We applied PBLR to five real scRNA-seq datasets and compared it with several clustering methods including SC3 (Kiselev et al., 2017), Seurat (Satija et al., 2015), SIMLR (Wang et al., 2017), and *k*-means on the first two t-SNE dimensions. The ratios of zeros of these datasets vary from 60.5% to 90.2% (Supplementary Table S5). On these datasets, PBLR and SC3 perform better and stable than other methods. PBLR exhibits the highest accuracy than other clustering methods on raw data except for Darmanis dataset (Figure 5A). On these datasets, the cluster membership has minor change after imputation by PBLR due to the better performance in identifying clusters in the first step of PBLR (Supplementary Figure S15).

Moreover, visualization of cells from Darmanis and Treutlein datasets using the raw data and the imputed data by PBLR, scImpute, and SAVER in the first two t-SNE components demonstrates that PBLR can make various cell sub-populations more separable. AT1 and AT2 cell sub-populations are clearly distinguishable using PBLR-imputed data. Clara cluster is separated from other ones, which is imputed by PBLR but masked by dropouts on raw data (Figure 5B). However, other two methods either separate cells from the same cluster into several small groups (scImpute) or cannot distinguish different clusters accurately (SAVER).

## Discussion

We present a powerful computational method for scRNA-seq data imputation. By case studies using available scRNA-seq data from diverse investigations and synthetic data simulated with a representative tool, we demonstrate that PBLR can reduce potential dropout events and biases by considering their sub-populations and observed expression distributions, and successfully derive biologically meaningful information from data imputation. Due to the high dimension of scRNA-seq data, dimension reduction is a powerful strategy for analyzing such data. However, some meaningful low-dimensional representations are masked by dropouts. PBLR can accurately remove the influence of dropouts in low dimensions on both synthetic and real datasets. Moreover, PBLR accurately recovers gene‒gene relationship which may be influenced by dropouts than other competing imputation methods.

One key component of PBLR is taking into account cell heterogeneity. Some imputation methods consider the cell heterogeneity such as scImpute, which detected similar cells based on cell‒cell distance. However, PBLR considers cell heterogeneity by first identifying the cell clusters using a custom method, which was shown to accurately capture the cell heterogeneity in comparison with other clustering methods such as SC3 and Seurat. There are also some other imputation methods that do not consider cell heterogeneity such as SAVER and ALRA. Both scImpute and PBLR select similar cells in advance to account for the heterogeneity of scRNA-seq data. However, SAVER imputes dropout values using the posterior mean and the prior estimated by other genes’ expression across all cells. To demonstrate the superior performance of this key component, we compare the performance of SAVER on synthetic dataset 2 with 10000 genes. When SAVER imputes the data with all cells, the various cell sub-populations are mixed together in the low-dimensional space. However, when SAVER imputes each cell sub-population individually, the three cell sub-populations are separated with only few mistakes (Supplementary Figure S2A). Therefore, considering the heterogeneity of scRNA-seq data may improve the accuracy of SAVER. Another key component of PBLR is taking into account structural effect of expression on dropout rate. scImpute divides genes for each cell into two groups A and B, where genes in A will be imputed based on genes in B of similar cells by non-negative least square regression. In PBLR, low-rank method solved by ADMM algorithm and truncated SVD regression across similar cells was used for approximating imputed matrix iteratively. Therefore, the variance of differential expression levels may be well imputed by PBLR. Moreover, PBLR considers the relationship between expected expression level and dropout rate of genes, and pulls back the imputed values if they deviate from this constraint. We quantified the consistence of the variation trend in differentially expressed genes between raw/imputed data and real full data using Spearman correlation, suggesting that imputed data by PBLR are more consistent with the real full data (Supplementary Figure S2B). As expected, considering the heterogeneity of cell sub-populations significantly improves the accuracy of SAVER in terms of the structural expression of differentially expressed genes (Supplementary Figure S2B, one-sided Wilcoxon rank-sum test, *P*-value = 7e−45). These results indicate that PBLR can not only recover low dimension representation, but also recover the variation in differential gene expression levels across cells.

PBLR consists of two main stages including identifying cell sub-populations and imputing dropouts. In the first stage, PBLR scales up well when the number of cells increases. In the second stage, singular value decomposition thresholding is the most time-consuming step. The computational efficiency will improve if feature selection and partial singular value decomposition method are used. Moreover, PBLR is an interactive method, cluster number and boundary function can be adjusted by users according to the characteristics of their datasets. Identifying cell sub-populations is a co-product of PBLR. Therefore, the utility of PBLR is very flexible that it can also be used to achieve a sub-population identification task. Comparison with existing clustering methods on real datasets demonstrates that PBLR also has more accurate clustering performance. Other efficient clustering methods in the first stage also can be chosen by the users. Here the cluster number is selected based on clustering stability. It definitely can be used if the cluster number is known in advance in some situations.

Taking together, PBLR can be used as a general method for addressing the dropout events prevalent in scRNA-seq data with the potential to reduce noise and correct biases. It serves as a proof of principle that bias can be removed by such a classical matrix recovery methodology with more practical considerations. Moreover, PBLR can be extended to impute data for other single-cell omics data by adapting its practical boundary observations. It provides a novel approach to omics data imputation, an area that is becoming increasingly important for improving big biological data in the single-cell biology era.

## Materials and methods

### Datasets and data preprocessing

The details of real datasets are shown in Supplementary Methods. For each dataset, genes expressed in <3 cells and cells with expressed genes <200 were removed. Then the data was normalized by a global method, i.e. expression of each gene was divided by the total expression for each cell, multiplied a scale factor (10000 by default) and log-transformed with pseudo-count 1.

### Gene selection

To account for technical noise in scRNA-seq data and select the informative genes, a set of highly variable genes was identified by calculating the average expression and Fano factor for each gene. We then bin the average expression of all genes into 20 evenly sized groups and normalize the Fano factor (Grun et al., 2014) within each bin. Genes with a larger normalized Fano factor value (0.05 by default) and its average expression being in predefined range (0.01‒3.5 by default) were selected. Moreover, genes with larger Gini index values (Jiang et al., 2016; Tsoucas and Yuan, 2018) can also be helpful to identify rare cell sub-populations (as used in Treutlein dataset).

### Sub-population and submatrix determination

The distance between each cell pair is computed by Pearson, Spearman, and Cosine metrics, respectively. These distance matrices (denoted by *D_k_*) are transformed to affinity matrices as follows: $A_{k}=e^{-D_{k}/\text{max}(D_{k})}$. We then apply SymNMF (Kuang et al., 2015) and INMF to the affinity matrices and raw scRNA-seq data to determine the consensus map, respectively (Supplementary Methods). Next, we adopt a consensus clustering method (Brunet et al., 2004) to identify cell sub-populations (Supplementary Methods). Finally, we get *g* cell sub-populations, and *g *+* *1 corresponding submatrices (*M*^(^^*k*^^)^, *k *=* *1, …, *g *+* *1) of the raw scRNA-seq data *M* by extracting the sub-matrix *M*^(^^*k*^^)^ (*k *=* *1, …, *g*) of each cell population of selected genes, and the sub-matrix *M*^(^^*g+*^^1)^ of the remaining genes across all cells. An optimal low rank *g* can be selected from a given range with the stability of clustering associated with each rank (Brunet et al., 2004).

The size of affinity matrix is *n* by *n*, where *n* is the number of cells. With the number of cells increasing, it will consume much memory. We focus more on accuracy than computational efficiency with the above strategy. Of course, the computational efficiency can be improved by GPU and other techniques. Here, we provided the following more efficient strategy as another option to consider cell heterogeneity on large datasets. We first construct a SNN graph by calculating the *k*-nearest neighbors (20 by default) for each cell on the data of the selected high variable genes. Then the fraction of SNNs between the cell and its neighbors is used as weights of the SNN graph. Next, we build the consensus matrix based on the clusters identified by applying the Leiden algorithm (Traag et al., 2019) to the constructed SNN graph with a range of resolution values (default: 0.1‒0.5 with the step equaling 0.1). After that, the cluster number *g* is determined according to the largest gap between singular values of the consensus matrix, which are computed by random singular value decomposition. Finally, the cell sub-populations are obtained by hierarchical clustering with the cluster number equaling *g*. PBLR with this strategy also performed well on the Shekhar dataset (Supplementary Figure S16).

### Boundary estimation

For the *k*th submatrix *M*^(^^*k*^^)^, we first compute the average expression *g_i_* of gene *i* in the observed space and the ratio of zeros *r_i_*. We only use the genes with *r_i_* being not equal to 0 and 1 because these genes either have no dropout (i.e. *r_i_* = 0) or are not expressed in all cells (i.e. *r_i_* = 1). After removing these genes, we estimate the upper boundary of gene *i* in the following ways. One way is to fit the ratio of zeros *r* vs. average expression level *g* with$r=e^{-\lambda g^{2}}$, and then the boundary of each gene is defined as the upper one-sided 95% confidence bound. However, we find that this exponential function does not fit well for some larger *r* and overestimate the boundary (Supplementary Figure S17). Therefore, we attempt to determine the boundary of gene *i* by introducing a piecewise function *U_i_*. First, to estimate the boundary of gene *i*, we define its neighbor gene set *S* = {*j* | |*r_j_–r_i_*|<*c*} using a radius *c* (default 0.05). Then, we compute the boundary of gene *i* by

$$U_{i}=\{\begin{array}{l} \min(g_{S}),\;\;\;\;\;r_{i}\geq 0.8\\ \max(g_{S}),\;\;\text{otherwise}, \end{array}$$

where $g_{S}=\{g_{j}|j\in S\}$ is the expression of the neighbor gene set. Moreover, we define a more sophisticated piecewise function,

$$U_{i}=\{\begin{array}{l} \min(g_{S}),\;\;\;\;\;\;\;\;\;\;r_{i}\geq 0.8\\ \text{quantile}(g_{S},0.25),\;\;\;0.6\leq r_{i}<0.8\\ \text{quantile}(g_{S},0.75),\;\;\;0.4\leq r_{i}<0.6\\ \max(g_{S}),\;\;\;\;\;\;\;\;\;\;\text{otherwise}. \end{array}$$

The sophisticated piecewise function is used as default. However, we also recommend choosing a proper boundary function by visually evaluating the scatter plot of ratio of zeros vs. average expression level on a sampled reference data (Supplementary Figure S17). We generate a reference data by dropping varying fractions (relevant to the dropout rate) of the gene measurements in the raw gene expression matrix. We simulate dropouts by setting true values to zero by sampling from a Bernoulli distribution using a dropout probability max (*p*_0_, 0.3), where *p*_0_ is the ratio of zeros in the raw expression matrix. By comparing the estimated boundary using both synthetic datasets and real datasets, our results indicate that the sophisticated piecewise function usually can give more accurate estimation. However, other two methods overestimate the boundary, especially for larger ratios of zeros (Supplementary FigureS17B and C).

### Bounded low-rank imputation algorithm

We adopt an ADMM algorithm (Gabay and Mercier, 1976; Chen et al., 2012) to solve the bounded low-rank matrix recovery model. Specifically, it can be reformulated as follows,

$$\begin{array}{c} \min_{X^{\left(k\right)}}{\left\|X^{\left(k\right)}\right\|}_{*}\\ s.t.\;X^{\left(k\right)}-Y=0\\ Y\in \{V|V_{\Omega }=M^{\left(k\right)},0\leq V_{\Omega^{⊥}}\leq U^{\left(k\right)}\}. \end{array}$$

The augmented Lagrangian function of the above function is

$$L(X^{\left(k\right)},Y,Z,\beta )={\left\|X^{\left(k\right)}\right\|}_{*}-\left\langle Z,X^{\left(k\right)}-Y\right\rangle +\frac{\beta }{2}{\left\|X^{\left(k\right)}-Y\right\|}_{F}^{2},$$

where *Z* is the Lagrange multiplier, β is the penalty parameter. We update the variables by alternatively updating *X*^(^^*k*^^)^, *Y*, *Z* as follows,

$$\{\begin{array}{l} Y^{t+1}=\arg\min_{Y\in V}L(X^{\left(k\right)}{}^{t},Y,Z^{t},\beta )\\ X^{\left(k\right)}{}^{t+1}=\arg\min L(X^{\left(k\right)},Y^{t+1},Z^{t},\beta )\\ Z^{t+1}=Z^{k}-\beta (X^{\left(k\right)}{}^{t+1}-Y^{t+1}) \end{array},$$

where *t* is the iteration index. In more detail, we can update variable *Y* by $\arg\min_{Y}\;L_{Y}=\beta /2{\left\|X^{(k)t}-Y\right\|}_{F}^{2}-\langle Z^{t},X^{(k)t}-Y\rangle$. Note that the partial derivative on *Y* of *L_Y_* is equal to $Z^{t}-\beta (X^{(k)t}-Y^{t})$, and thus it can be reformulated as $\langle \begin{array}{cc} Y-Y^{t+1}, & Y^{t+1}+1/\beta Z^{t}-X^{(k)t} \end{array}\rangle \geq 0,\forall Y\in V$. The solution is $Y^{t+1}=P_{V}[X^{(k)t}-1/\beta Z^{t}]$, where $P_{V}$ is the projection operator onto *V* space. The solution can be written as follows,

$$Y^{t+1}=\{\begin{array}{l} M_{ij},\text{if}\;(i,j)\in \Omega \\ 0,\text{if}\;(i,j)\in \Omega^{⊥},\;B^{t+1}(i,j)<0\\ U_{ij},\text{if}\;(i,j)\in \Omega^{⊥},{\;B}^{t+1}(i,j)>U_{ij}^{\left(k\right)}\\ B_{ij}^{t+1},\text{otherwise} \end{array},$$

where $B^{t+1}=X^{(k)t}-1/\beta Z^{t}$. Then let $A^{t+1}=Y^{t+1}+1/\beta Z^{t}$, and $A^{t+1}=V_{1}^{t+1}\Sigma^{t+1}V_{2}^{t+1},$where $\Sigma^{t+1}=\text{diag}(\sigma_{1}^{t+1},\sigma_{2}^{t+1},\ldots ,\sigma_{r^{t+1}}^{t+1})$ and $\sigma_{j}^{t+1}$ is the eigenvalues of $A^{t+1}$. According to a traditional solution in previous studies (Cai et al., 2010; Ma et al., 2011), the update rule for *X* is $X^{t+1}=V_{1}^{t+1}{\hat{\Sigma }}^{t+1}V_{2}^{t+1},$ where ${\hat{\Sigma }}^{t+1}=\text{diag}\{{\left(\sigma_{j}^{t+1}-1/\beta \right)}_{+}\}$. Therefore, we only need to compute the eigenvalues larger than 1/β and we use PROPACK package to compute the partial SVD. Previous studies (Glowinski, 1984; Glowinski and Le Tallec, 1989) have proved that the step for updating the Lagrange multiplier can be generalized into $Z^{t+1}=Z^{t}-\gamma \beta (X^{(k)t+1}-Y^{t+1}),0<\gamma <\sqrt{5}+1/2$. In the proposed algorithm, we use the same parameter γ=1.6 and β=2.5mn as in a previous study (Chen et al., 2012). This procedure is summarized in Algorithm 1 (Supplementary Methods).

### PBLR algorithm

The whole procedure for solving scRNA-seq imputation is summarized in Algorithm 2 (Supplementary Methods).

### Imputation accuracy evaluation on synthetic datasets

To quantify the difference between imputed data and full data, we calculate two measures: SSE and PCC. SSE is defined as $\text{SSE}=\sum_{i}\sum_{j}{\left(F_{ij}-X_{ij}\right)}^{2}$, where *F_ij_* represents the real expression of gene *i* in cell *j*, and *X_ij_* represents the corresponding imputed value. PCC is computed between each column pair (*F._j_* and *X._j_*) in *F* and *X*.

### NMI

The true partition of *m* clusters and the inferred partition given by PBLR are denoted by *U* = {*U_1_*, …, *U_m_*} and *V* = {*V_1_*, …, *V_n_*}, respectively. Then the NMI is defined as NMI=2I(U,V)/H(U)+H(V), where *I*(*U*, *V*) is mutual information, *H*(*U*) is the entropy of partition *U*.

### POS

To measure the accuracy of the reconstructed pseudotime, we define POS = *C*/(*N_c_ + C*), where *C* and *N_c_* represent the number of concordant and disconcordant pairs of cells between the inferred pseudotime and golden standard (e.g. true data collection time), respectively.

### Manifold preservation score

The manifold preservation score is defined to quantify the similarity of each cell’s neighborhood in a low-dimensional space obtained from the full data without dropouts vs. the imputed data of each method (Welch et al., 2019). We first apply a dimension reduction method to the full data and the imputed data respectively, then build a *k*-nearest neighbor graph in each low-dimensional space, and finally count how many of each cell’s nearest neighbors in the low-dimensional space of full data are also nearest neighbors in the low-dimensional space of imputed data. High manifold preservation score indicates the well preservation of manifold distance in the imputed data compared to that in the full data.

### Data access

Deng, Darmanis, Treutlein, and Zeisel datasets can be obtained from Gene Expression Omnibus (GEO) with GSE45719, GSE6785, GSE52583, and GSE60361, respectively. Pollen dataset is available at Sequence Read Archive with SRP041736. HEE, MEF, and Campbell datasets can be obtained from GEO with GSE36552, GSE67310, and GSE90806, respectively. Shekhar data are available from https://github.com/broadinstitute/BipolarCell2016. The package of PBLR is available at https://github.com/amsszlh/PBLR or http://page.amss.ac.cn/shihua.zhang/software.html.

## Supplementary material

Supplementary material is available at *Journal of Molecular Cell Biology* online.

## Funding

This work was supported by the National Key R&D Program of China (2019YFA0709501), the National Natural Science Foundation of China (11661141019 and 61621003), the National Ten Thousand Talent Program for Young Top-notch Talents, the CAS Frontier Science Research Key Project for Top Young Scientist (QYZDB-SSW-SYS008), and Shanghai Municipal Science and Technology Major Project (2017SHZDZX01).

**Conflict of interest**: none declared.

## Supplementary Material

mjaa052_Supplementary_Data
