# Supplementary material for: Imputing single-cell RNA-seq data by considering cell heterogeneity and prior expression of dropouts
Source: J Mol Cell Biol. 2020 Oct 1;13(1):29–40. doi: 10.1093/jmcb/mjaa052 (PMC8035992; doi:10.1093/jmcb/mjaa052)
Supplement: mjaa052_Supplementary_Data [file mjaa052_supplementary_data.pdf]

Supplemental Material for

Imputing single cell RNA-seq data by considering cell heterogeneity and prior expression level of dropouts

Lihua Zhang[1,2] and Shihua Zhang[1,2,3,4*]

[1]NCMIS, CEMS, RCSDS, Academy of Mathematics and Systems Science, Chinese Academy of Sciences, Beijing 100190, China;
[2]School of Mathematical Sciences, University of Chinese Academy of Sciences, Beijing 100049, China;
[3]Center for Excellence in Animal Evolution and Genetics, Chinese Academy of Sciences, Kunming 650223, China;
[4]Key Laboratory of Systems Biology, Hangzhou Institute for Advanced Study, University of Chinese Academy of Sciences, Chinese Academy of Sciences, Hangzhou 310024, China.

[*]**Email**: zsh@amss.ac.cn

Supplementary Methods

Datasets

We evaluated the performance of PBLR in imputing scRNA-seq data on both simulated and real datasets. The simulated datasets were generated using Splatter (Zappia et al., 2017), and the parameters used are shown in Supplemental Material Table S1. The four real datasets include two small datasets collected from human and mouse embryonic development, and two large-scale datasets (~20k cells). HEE dataset (Yan et al., 2013) consists of 88 cells from seven stages (from oocytes to blastocyst) during human early embryo (HEE) development. We obtained a data matrix with 16658 genes across 88 cells after filtering out genes that were expressed in less than 5 cells. MEF dataset (Treutlein et al., 2016) describes the reprogramming from mouse embryonic fibroblasts (MEFs) to induce neuronal (iN) cells. To reconstruct the reprogramming path from MEFs to iN cells, similar to the original study (Treutlein et al., 2016), we used 221 cells collected at multiple time points (0, 2, 5, 22 days) after removing cells that appeared stalled in reprogramming due to Ascl1 silencing or cells converging on the alternative myogenic fate.

In mouse retinal dataset, ~28,000 cells were profiled from a transgenic mouse line that marks bipolar cells (BCs) of the mouse retina (Shekhar et al., 2016). We obtained the digital expression matrix of 13,166 appreciably expressed genes across 27,499 cells using the R Markdown Code (https://github.com/broadinstitute/BipolarCell2016). 18 cell clusters, including 13 cone BC clusters, 1 rod bipolar cell cluster and 4 non-BC clusters, were obtained in the original study. In Campbell dataset, 20,921 cells were profiled from acutely dissociated Arc-ME cells of adult mice under six feeding conditions: ad libitum access to standard mouse

chow, low-fat diet or high-fat diet, as well as overnight fasting, with or without subsequent refeeding(Campbell et al., 2017). 20 distinct clusters (including neuron and non-neuron cell types) was identified in the original study. To visualize cells in the low dimensional space, we used Seurat package and performed batch effect correction (using Combat), following the same procedure as the original study. As batch effect correction will totally change the proportion of zeros in the raw digital expression matrix, we thus used the raw expression matrix as an input of various imputation methods. We performed batch correction on the imputed data by scImpute and SAVER, and then run Seurat pipeline to project cells onto t-SNE space. We did not perform batch correction on the PBLR-imputed data because we did not observe explicit batch effects after projecting cells onto t-SNE space.

To evaluate the performance of PBLR in identifying cell subpopulations, we adopted five real datasets (Supplemental Material Table S5). Deng dataset (Deng et al., 2014) consists of 22431 genes across 268 cells, which was taken from the mouse embryo development process from zygote to blastocyst. Pollen dataset (Pollen et al., 2014) contains 301 single cells across diverse tissues, including neural cells and blood cells. This dataset was used to test the utility of low-coverage scRNA-seq to identify cell subpopulations. Darmanis dataset (Darmanis et al., 2015) was used to capture the cellular complexity of the adult and fetal human brain, including 20214 genes across 90 cells. These cells were divided into six groups, including astrocytes, endothelial, microglia, neurons, fetal quiescent and fetal replicating. Zeisel dataset (Zeisel et al., 2015) contains 3005 single cells came from mouse cortex and hippocampus. The cells were collected by unique molecule identifier (UMI) and divided into nine clusters. Treutlein dataset (Treutlein et al., 2014) was taken from distal mouse lung epithelial cells at different developmental stages. We used 80 single cells at E18.5 stage, which were clustered into five groups including BP, AT1, AT2, Clara and Ciliated.

Symmetric non-negative matrix factorization (SymNMF)

SymNMF decomposes a non-negative affinity matrix into two symmetric non-negative low-rank matrices as follows,

$$\begin{aligned} &\min_H \|A - HH'\|_F^2 \\ &\text{s.t.} \quad H \geq 0, \end{aligned}$$

where A is the affinity matrix and H is the non-negative low-rank matrix, which can be used to indicate clustering assignment. As SymNMF is a non-convex problem that may lead to the assignment being not unique, we repeat it 20 times with random initial values.

Incomplete non-negative matrix factorization (INMF)

Let M_s represent the raw expression matrix with selected genes as its rows and cells as its columns. Let S represent the indicator matrix with element $S(i,j)$=1 if $M_s(i,j)$ is a non-zero

value, otherwise *S(i,j)*=0. The following INMF model is used to learn a low-rank coefficient matrix H_s to assign each cell to one cluster,

$$\begin{aligned} &\min_{W_s, H_s} \|S \odot (M_s - W_s H_s)\|_F^2 \\ &\text{s.t.} \quad W_s, H_s \geq 0, \end{aligned}$$

where $\odot$ is dot product. Similar to SymNMF, we also repeat INMF 20 times with random initial values. SymNMF and INMF are solved by alternative nonnegative least square and multiplier update algorithm, respectively.

Consensus clustering method

Each column's maximum value of *H* or H_s obtained from SymNMF or INMF under each run is used to determine the cluster membership (Kim and Tidor, 2003). The membership can be represented by a connectivity matrix *C*, with element *C(i,j) = 1* if cell *i* and cell *j* are assigned into the same cluster, otherwise *C(i,j) = 0*. Then the connectivity matrices are summed across all runs and normalized by the number of runs. Thus, we obtain a consensus matrix $\bar{C}$ and the entries vary from 0 to 1. The entry represents the probability of cells being grouped together. Next, hierarchical clustering (HC) with average linkage is applied on ***1***-$\bar{C}$, where ***1*** is matrix with all entries equaling 1. The clustering stability can be estimated by the cophenetic correlation coefficient ρ, which is computed as the Pearson correlation of ***1***-$\bar{C}$ and the distance between cells inferred by average linkage. We found that the number of clusters (in a reasonable range) had minor effect on the performance (Supplemental Material Fig S18). Let ρ_1 represent coefficient obtained from the average consensus matrix of Pearson, Spearman and Cosine distance, and ρ_2 stands for that from the consensus matrix computed from INMF. If $|\rho_1 - \rho_2| > cutoff$, the final clustering result is computed by the average linkage HC on ***1***-$\bar{C}^{\max}$, where $\bar{C}^{\max}$ means the consensus matrix of the lager coefficient. If $|\rho_1 - \rho_2| \leq cutoff$, the final clustering result is computed on ***1***-$\bar{C}^{avg}$, where $\bar{C}^{avg}$ is the average of all consensus matrices.

Bounded low-rank imputation algorithm

Algorithm 1: BLR

- Step 1: Initialize X^t, Z^t with zero matrices, $\gamma = 1.6$, $\beta = 2.5/\sqrt{mn}$, tol = 10^{-6} and set the iteration step *t=0*.
- Step 2: Fix X^t, Z^t and update Y^{t+1} with

$$Y^{t+1} = \begin{cases} M_{ij}, \text{if } (i,j) \in \Omega \\ 0, \textit{if } (\text{i,j}) \in \Omega^{\perp}, B^{t+1}(i,j) < 0 \\ U_{ij}, \textit{if } (\text{i,j}) \in \Omega^{\perp}, \text{B}^{t+1}(i,j) > U_{ij} \\ B_{ij}^{t+1}, \text{otherwise} \end{cases},$$

- Step 3: Fix Z^t, Y^{t+1}, update X^{t+1} via the well-known singular value shrinkage by

$(V_1, S, V_2) = svd(Y^{t+1} + \frac{1}{\beta} Z^t)$, $X^{t+1} = V_1 \mathbb{S}_{1/\beta}[S] V_2^T$.

- Step 4: Fix X^{t+1}, Y^{t+1}, update Z^{t+1} by $Z^{t+1} = Z^t - \gamma\beta(X^{t+1} - Y^{t+1})$.
- Step 5: Let $t \leftarrow t+1$, repeat Steps 2-4 until the following convergence criterion is satisfied: $\frac{\|X^{t+1} - X^t\|_F}{\|X^t\|_F} < \text{tol}$.

PBLR algorithm

Algorithm 2: PBLR

- Step 1: Input raw data M, cluster number K, outer iterations N, threshold c.
- Step 2: Data filtering and normalization.
- Step 3: Select highly variable genes, and M_S represents the sub-matrix with selected genes across cells. Compute cell-cell distance matrices based on Pearson, Spearman and Cosine metrics, then transform to affinity matrices.
- Step 4: Run SymNMF 20 times on each affinity matrix and compute average consensus matrix C_1 and ρ_1.
- Step 5: Run INMF 20 times on M_S and compute consensus matrix C_2 and ρ_2.
- Step 6: If $|\rho_1 - \rho_2| > c$, suppose $\rho_k = \max(\rho_1, \rho_2)$, then determine cell clustering assignment by average linkage HC on $\mathbf{1}$-C_k, else determine clustering result by average linkage HC on $\mathbf{1}$-C, where C is the average matrix of C_1 and C_2.
- Step 7: Let $M_s^{(k)}$ and $M_r^{(K+1)}$ represent the gene expression of selected genes across the k-th subpopulation and remaining genes across all cells. Obtain the imputed sub-matrices by **Algorithm 1**, respectively.
- Step 8: Integrate these imputed sub-matrices to form the output data matrix.

References

Campbell, J.N., Macosko, E.Z., Fenselau, H., et al. (2017). A molecular census of arcuate hypothalamus and median eminence cell types. Nat Neurosci *20*, 484-496.

Darmanis, S., Sloan, S.A., Zhang, Y., et al. (2015). A survey of human brain transcriptome diversity at the single cell level. P Natl Acad Sci USA *112*, 7285-7290.

Deng, Q.L., Ramskold, D., Reinius, B., et al. (2014). Single-cell RNA-seq reveals dynamic, random monoallelic gene expression in mammalian cells. Science *343*, 193-196.

Kim, P.M., and Tidor, B. (2003). Subsystem identification through dimensionality reduction of large-scale gene expression data. Genome Res *13*, 1706-1718.

Pollen, A.A., Nowakowski, T.J., Shuga, J., et al. (2014). Low-coverage single-cell mRNA sequencing reveals cellular heterogeneity and activated signaling pathways in developing cerebral cortex. Nat Biotechnol *32*, 1053-1058.

Shekhar, K., Lapan, S.W., Whitney, I.E., et al. (2016). Comprehensive classification of retinal bipolar neurons by single-cell transcriptomics. Cell *166*, 1308-1323.

Treutlein, B., Brownfield, D.G., Wu, A.R., et al. (2014). Reconstructing lineage hierarchies of the distal lung epithelium using single-cell RNA-seq. Nature *509*, 371-375.

Treutlein, B., Lee, Q.Y., Camp, J.G., et al. (2016). Dissecting direct reprogramming from fibroblast to neuron using single-cell RNA-seq. Nature *534*, 391-395.

Yan, L., Yang, M., Guo, H., et al. (2013). Single-cell RNA-seq profiling of human preimplantation embryos and embryonic stem cells. Nat Struct Mol Biol *20*, 1131-1139.

Zappia, L., Phipson, B., and Oshlack, A. (2017). Splatter: simulation of single-cell RNA sequencing data. Genome Biol *18*, 174.

Zeisel, A., Munoz-Manchado, A.B., Codeluppi, S., et al. (2015). Cell types in the mouse cortex and hippocampus revealed by single-cell RNA-seq. Science *347*, 1138-1142.

Supplementary Figures

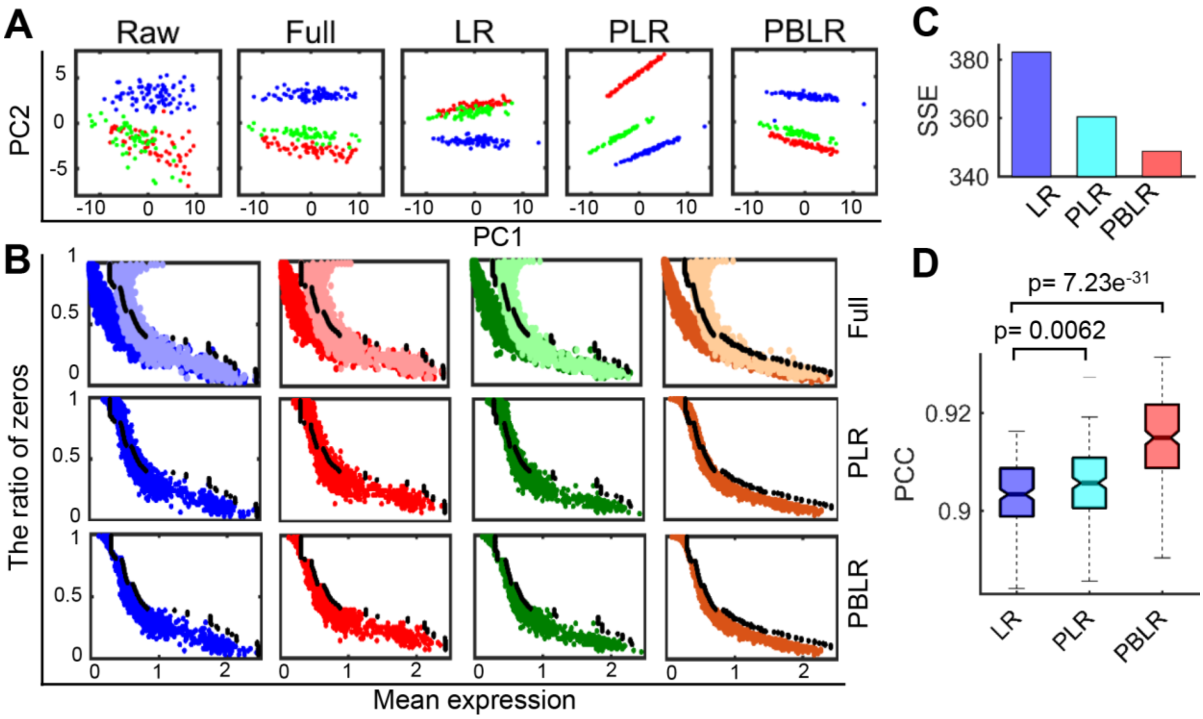

Figure S1. Comparison of PBLR with LR and PLR on the synthetic dataset 1 with three sub-populations. (A) PCA visualization of the raw data, full data (data without dropouts) as well as imputed ones by LR, PLR and PBLR, respectively. LR represents the typical low-rank matrix recovery method, PLR indicates the population-based LR method. Visualization by PCA on the full data (data without dropouts) clearly shows three separated subpopulations or clusters. However, the clusters are confounded on the raw data due to the existence of dropouts. We applied LR to impute the raw data and revealed mixed clusters (subpopulations) in the PCA space. Interestingly, performing LR on the inferred sub-matrices determined by cell sub-populations (denoted as PLR) can well separate them with more disperse clusters than those in the full data. However, it tends to over-estimate the expression of low-expressed genes compared to the real expression levels (see panel B). Based on PLR, by further taking expression upper boundary into account, PBLR imputed data shows well separated clusters and more consistent distributions to the full data in the low-dimensional space as well as more reasonable expression-to-dropout relationships (see panel B). **(B)** Scatter plots of each gene with x axis representing log-transformed mean gene expression value and y axis representing the ratio of zeros across cells of each group. The top row shows distribution of real values of full data in the zero space (dark color) and non-zero space (light color) respectively for each sub-matrix. The middle and bottom rows show that of imputed values by PLR and PBLR for each sub-matrix respectively. Dots in different colors stand for imputed values of each sub-matrix in the zero space. The black dots represent the upper boundary. **(C)** SSE computed between the full data and the imputed ones by LR, PLR and PBLR respectively. **(D)** PCC computed for all single cell pair between the full data and the imputed ones by LR, PLR and PBLR respectively. *P*-value is computed by one-side Wilcoxon rank-sum test. As expected, compared to LR and PLR, PBLR gives more accurate imputed values in terms of sum of squared error (SSE) and Pearson correlation coefficients (PCC).

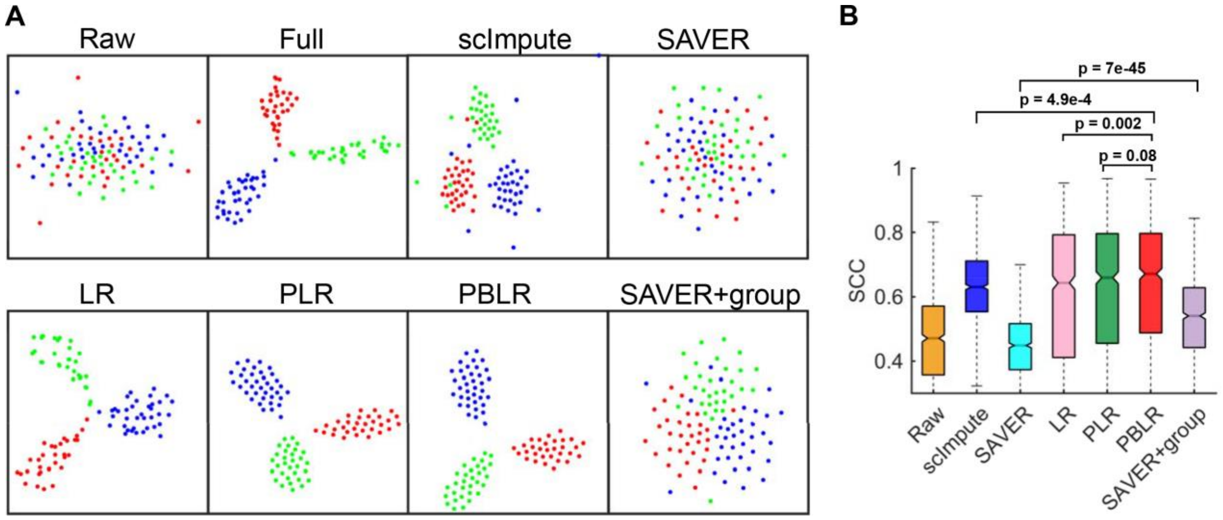

Figure S2. **Illustration of the key components of PBLR. (A)** Visualization of cells by the first two t-SNE components on the raw synthetic dataset 2 with a large number of genes (n = 10,000), full data and imputed ones by scImpute, SAVER, LR, PLR, PBR and SAVER in each group respectively. The large-scale synthetic dataset 2 was simulated using the same parameter with *dropout.shape* = -0.05 and the number of genes being 10,000 (Table S1). **(B)** The Spearman correlation coefficient (SCC) of differential genes across all cells between full data and raw or imputed data.

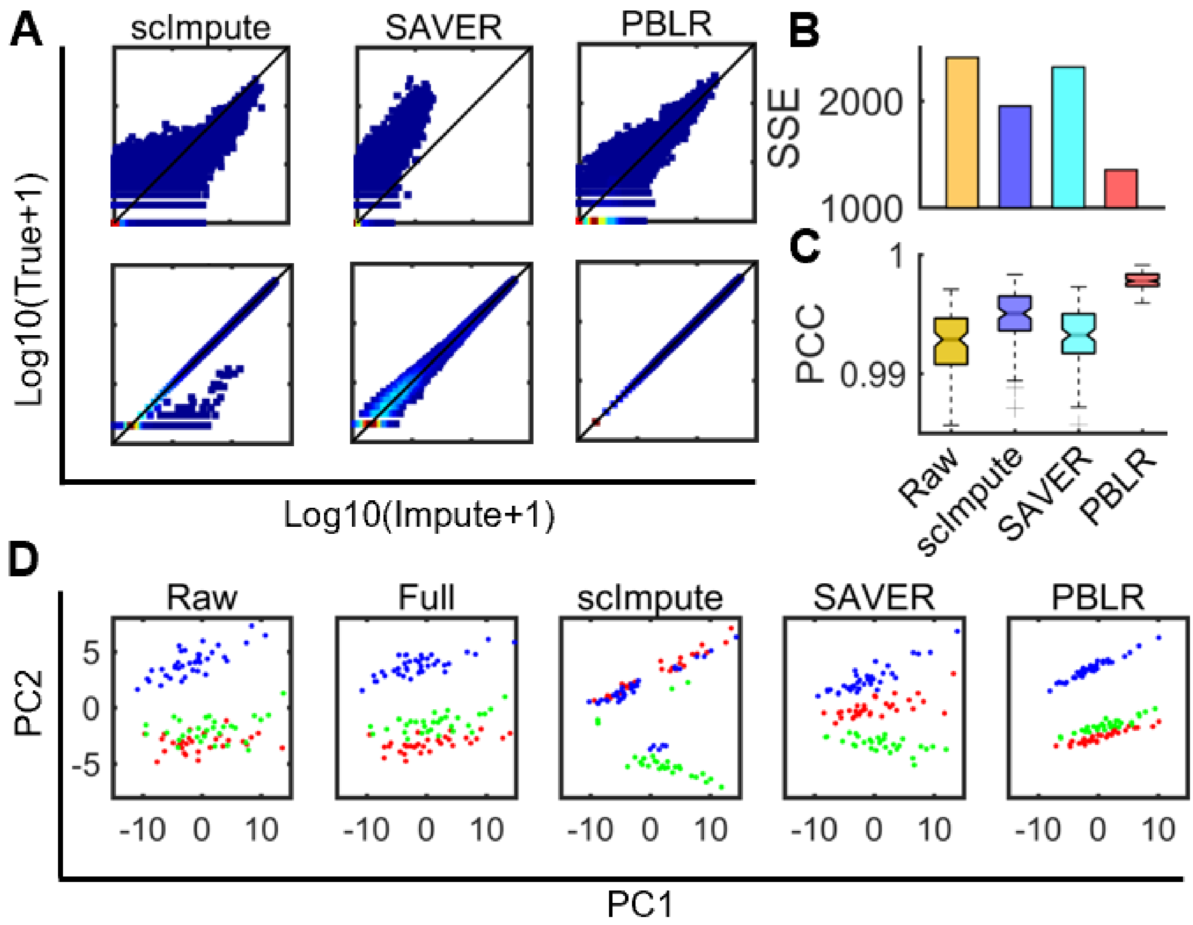

Figure S3. Comparison of the imputation performance of scImpute, SAVER and PBLR on synthetic datasets 3. (A) Density plot of the imputed values versus real ones in the zero space (top) and the observed non-zero space (bottom), respectively. In the non-zero space, scImpute treats many moderate expression values as dropouts and imputes them by larger values than the true ones, while SAVER recovers non-zero values with some deviations. **(B)** SSE values computed between the full data and the raw data as well as the imputed ones. **(C)** PCC values of all single cell pair computed between the full data and the raw data as well the imputed ones. All of these three methods decrease the SSE values and improves PCC values relative to that of the raw data. However, PBLR gives more accurate imputed data than other two methods in terms of SEE and PCC values. **(D)** PCA plot on raw data, full data, and imputed data matrices by scImpute, SAVER, PBLR, respectively. The three cell clusters are distinguishable on the full data although the red and green clusters are close to each other, but they become less well separated in the raw data with dropout events. However, the relationships among these three clusters are separated after we applied PBLR. For other two methods, scImpute cannot separate the red and blue clusters. Although SAVER can distinguish these three clusters, it changes the data distribution as the relative distances between the three clusters are very different to that of the full data.

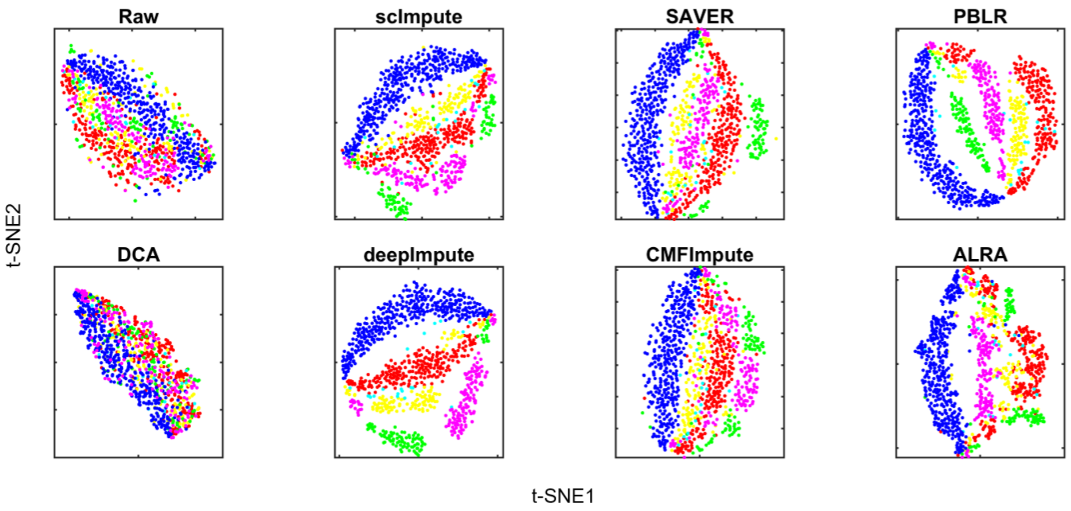

Figure S4. tSNE visualization of the reduced dimensions of raw data and imputed data of 7 imputation methods on synthetic dataset 4.

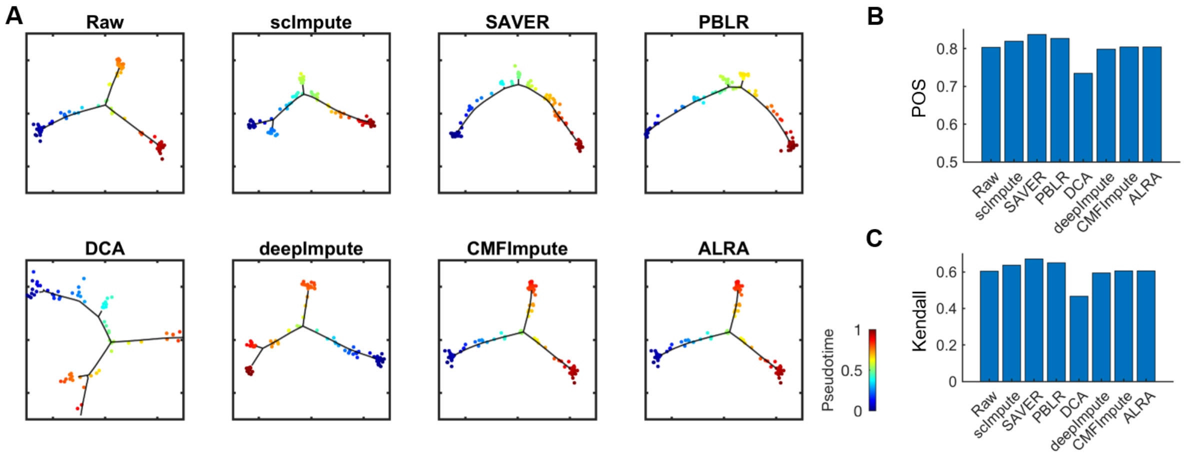

Figure S5. Performance of reconstructing pseudotime order of 7 imputation methods on synthetic dataset 5. (A) Visualization of the inferred trajectory by Monocle on raw and imputed data of 7 imputation methods. **(B)** Bar plots of POS scores between pseudotime inferred by Monocle and golden standard pseudotime. **(C)** Bar plots of Kendall's rank correlation coefficients between pseudotime inferred by Monocle and golden standard pseudotime.

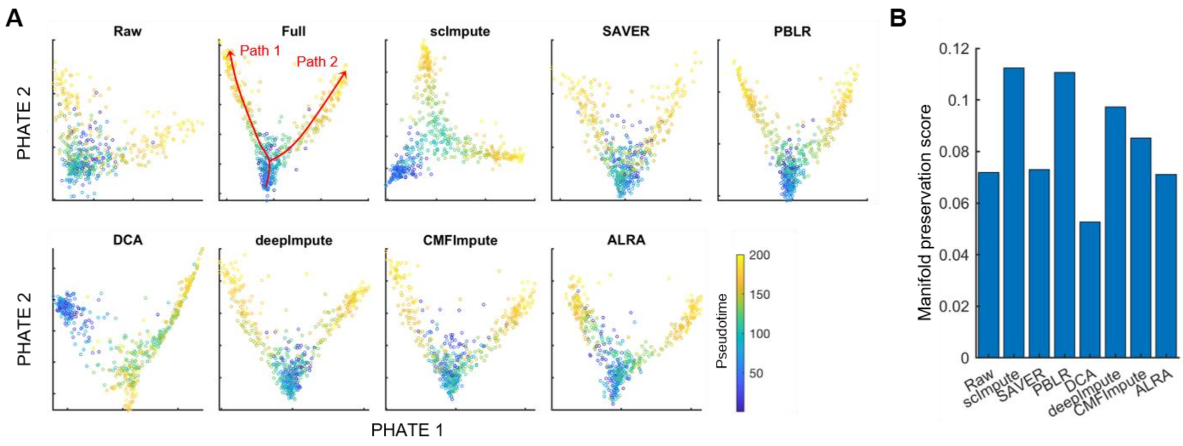

Figure S6. Imputation performance on synthetic dataset 6 describing a continuous cell trajectory with two paths. (A) Visualization of cells on the first two PHATE components using raw data, full data (golden standard data without dropout) and imputed data of eight methods. Cells were colored with golden standard steps (i.e., pseudotime). **(B)** Comparison of manifold preservation scores between full data and raw data or imputed data. High manifold preservation score indicates the well preservation of manifold distance in the imputed data compared to that in the full data.

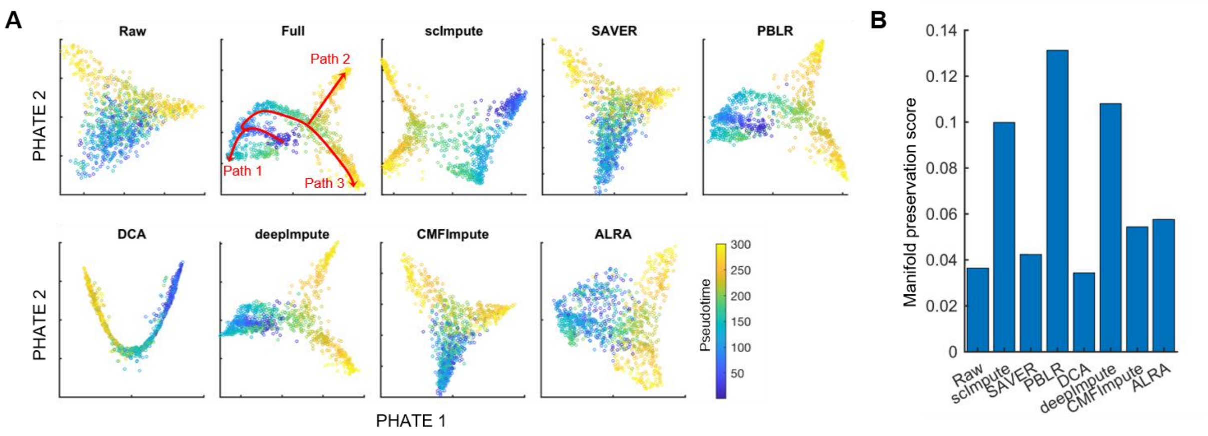

Figure S7. Imputation performance on synthetic dataset 7 describing a continuous cell trajectory with three paths. (A) Visualization of cells on the first two PHATE components using raw data, full data (golden standard data without dropout) and imputed data of eight methods. Cells were colored with golden standard steps (i.e., pseudotime). **(B)** Comparison of manifold preservation scores between full data and raw data or imputed data.

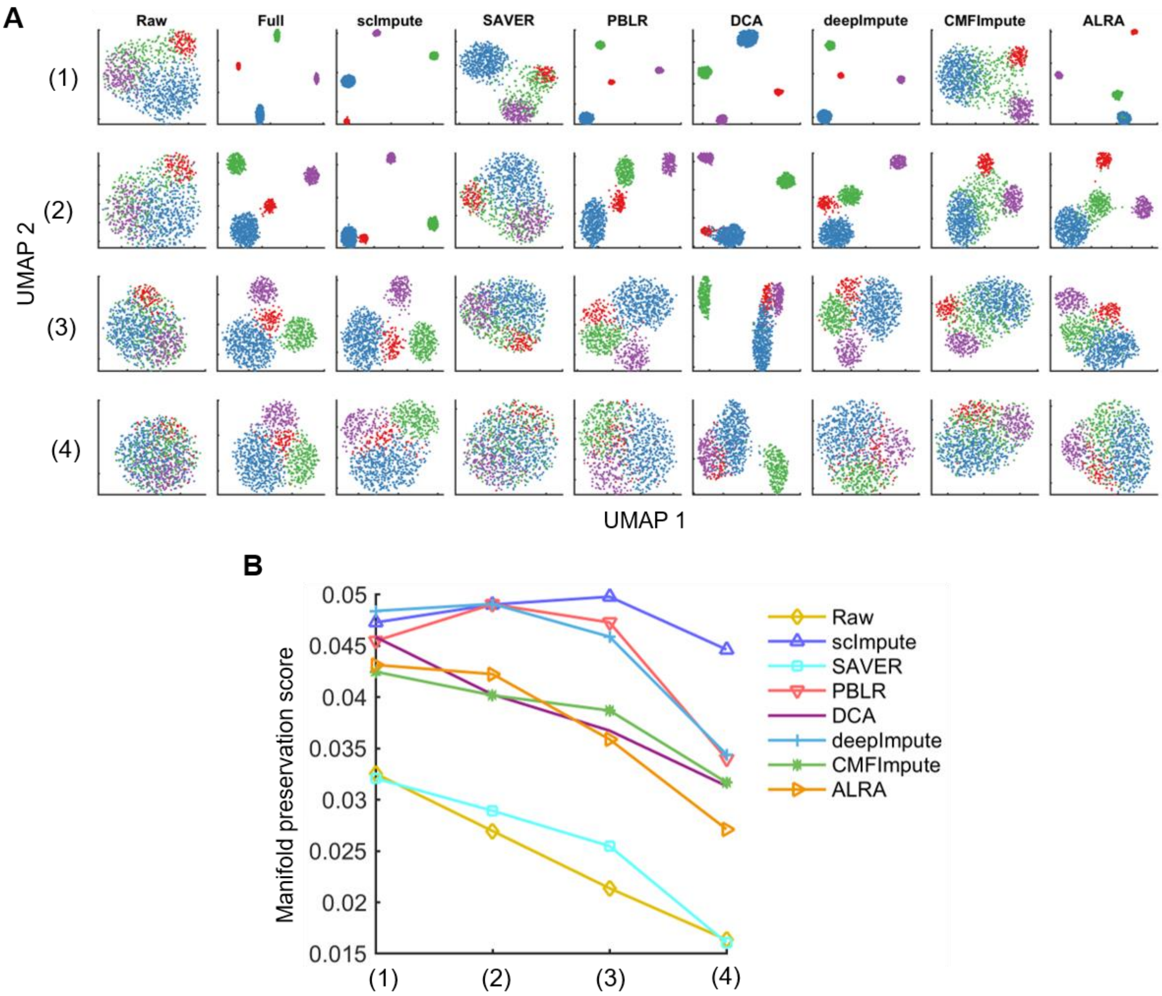

Figure S8. Imputation performance on synthetic dataset 8 with varied cell subpopulation distance and degree of noise. (A) Visualization of cells on the first two UMAP components using raw data, full data (golden standard data without dropout) and imputed data of eight methods. Cells were colored with golden standard cell subpopulation labels. Each row represents one data corresponding to one pair of parameters. The cell subpopulation distance decrease and the degree of noise increase from data (1) to (4), which are controlled by the parameter de.facLoc varying from 4 to 11, and the parameter bcv.common varying from 0.1 to 0.44 in the Splatter package, respectively. **(B)** Comparison of manifold preservation scores between full data and raw data or imputed data. scImpute, PBLR and deepImpute consistently had higher scores than other methods, suggesting the better preservation of cell-cell distances in the imputed data.

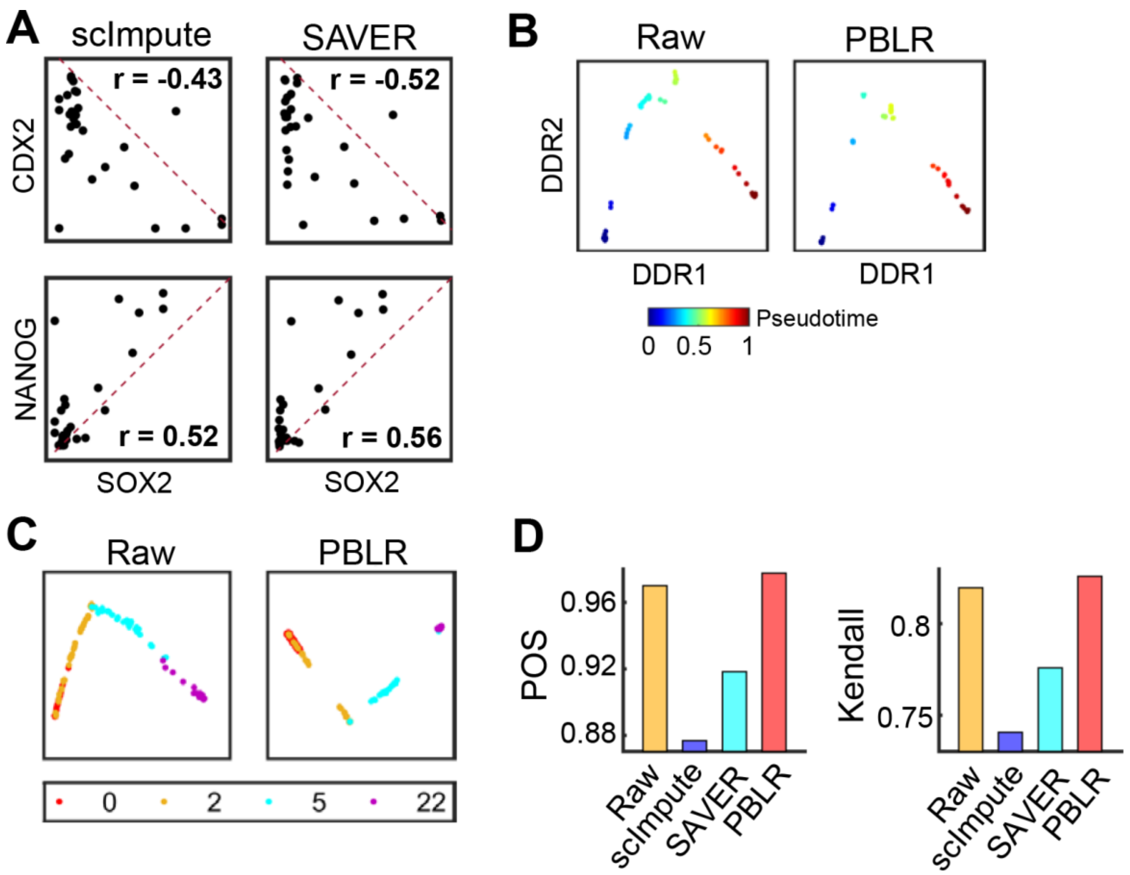

Figure S9. Comparison of the performance of scImpute, SAVER and PBLR on inferring pseudotime on two real datasets. (A) Scatter plots of marker genes' expression in imputed HEE data by scImpute and SAVER. The corresponding Spearman correlation coefficient (SCC) of expression values in the late blastocyst cells is shown. **(B)** Visualization of the inferred trajectory on HEE raw data and PBLR imputed data in the first two discriminative dimensions computed by Monocle 2. Each dot represents a cell, which is colored by the inferred pseudotime. Cells with higher values are in more differentiated states. **(C)** Visualization of the inferred trajectory on MEF raw data and PBLR imputed one in the first two discriminative dimensions computed by Monocle 2. Each dot represents a cell colored by the experimental time points. **(D)** Barplots of POS scores and Kendall's rank correlation coefficients (quantifying the similarity between the inferred pseudotime and the real experimental time points) after applying Monocle 2 on MEF data and imputed one by scImpute, SAVER and PBLR, respectively.

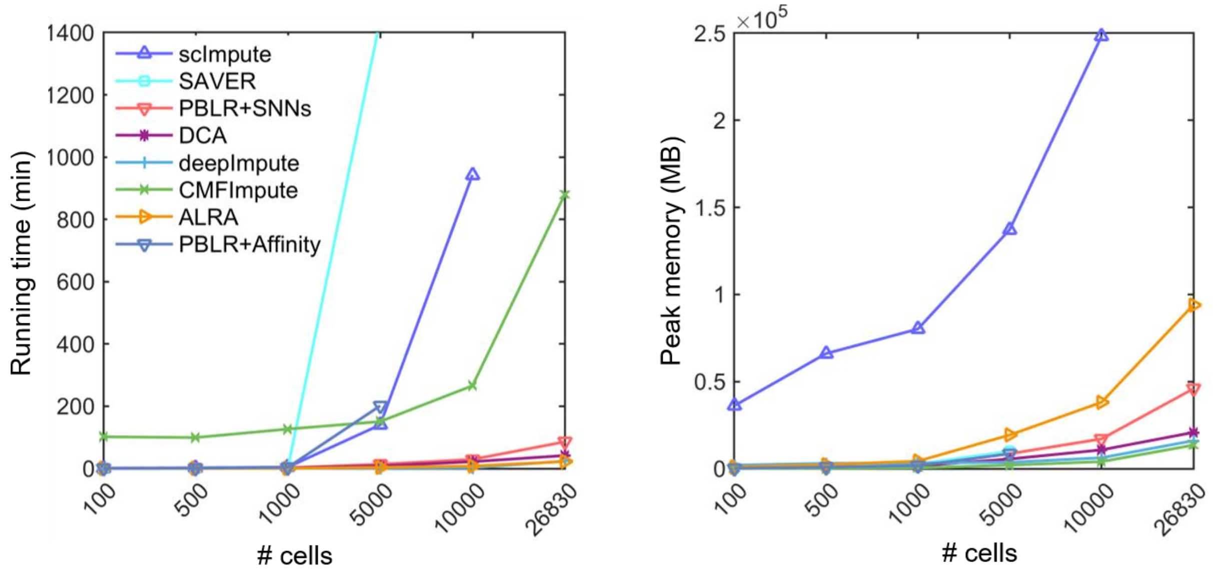

Figure S10. Evaluation running time (left, y-axis is minutes) and peak RAM usage for Shekhar dataset with different cell numbers. PBLR+SNNs represents PBLR with second strategy of cell heterogeneity consideration, while PBLR+affinity represents PBLR with the first strategy of cell heterogeneity consideration.

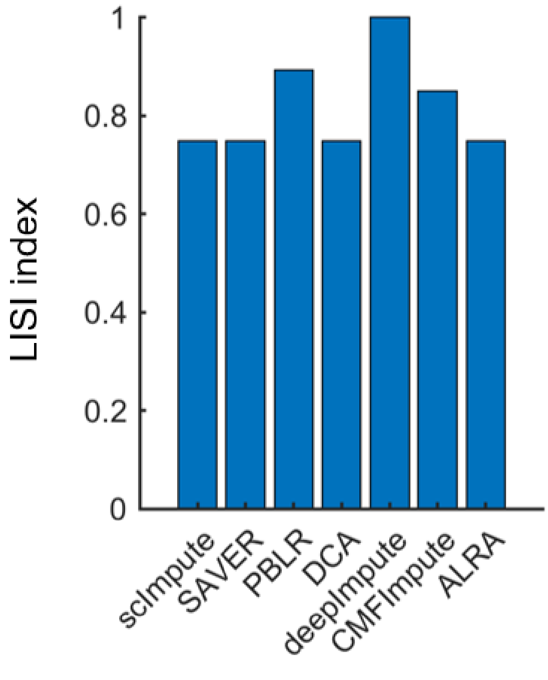

Figure S11. Quantitative evaluation of batch effect correction using local inverse Simpson's index (LISI) metric on imputed Shekhar data by 7 imputation methods. For comparison between methods, we took the median value of the scores computed for all cells in the dataset, and scaled such that 0 and 1 denote the worst and best possible scores respectively.

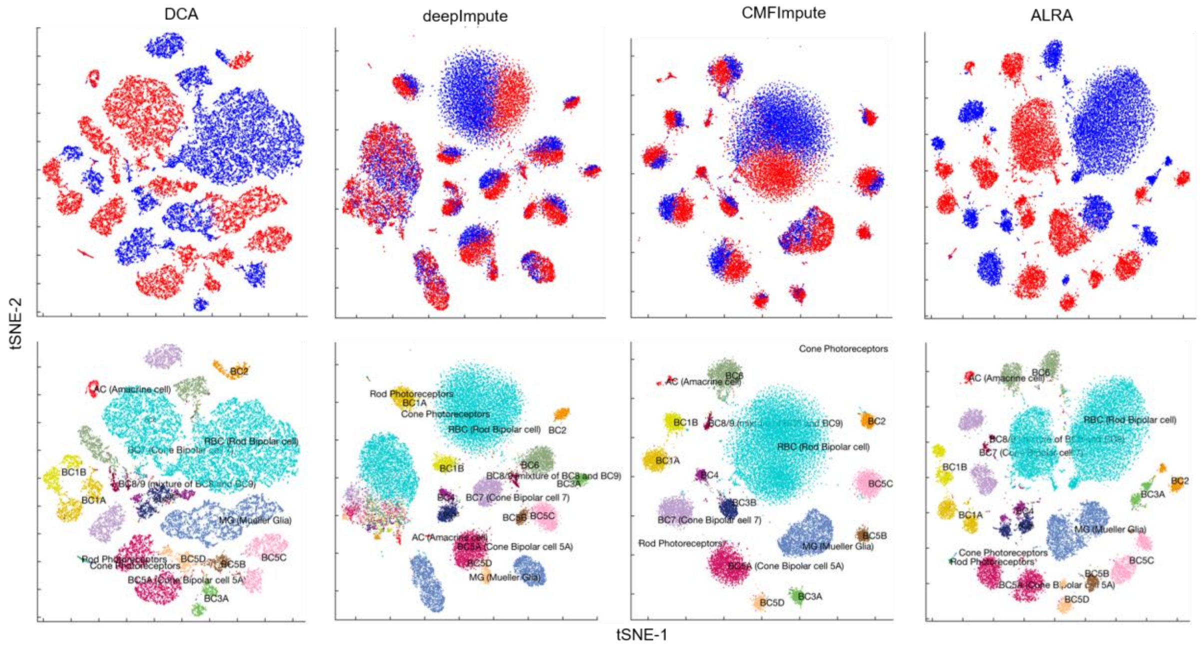

Figure S12. Cells are visualized on the first two t-SNE components using the imputed Shekhar data by DCA, deepImpute, CMFImpute and ALRA. Cells are colored by batches (top) and cell types (bottom).

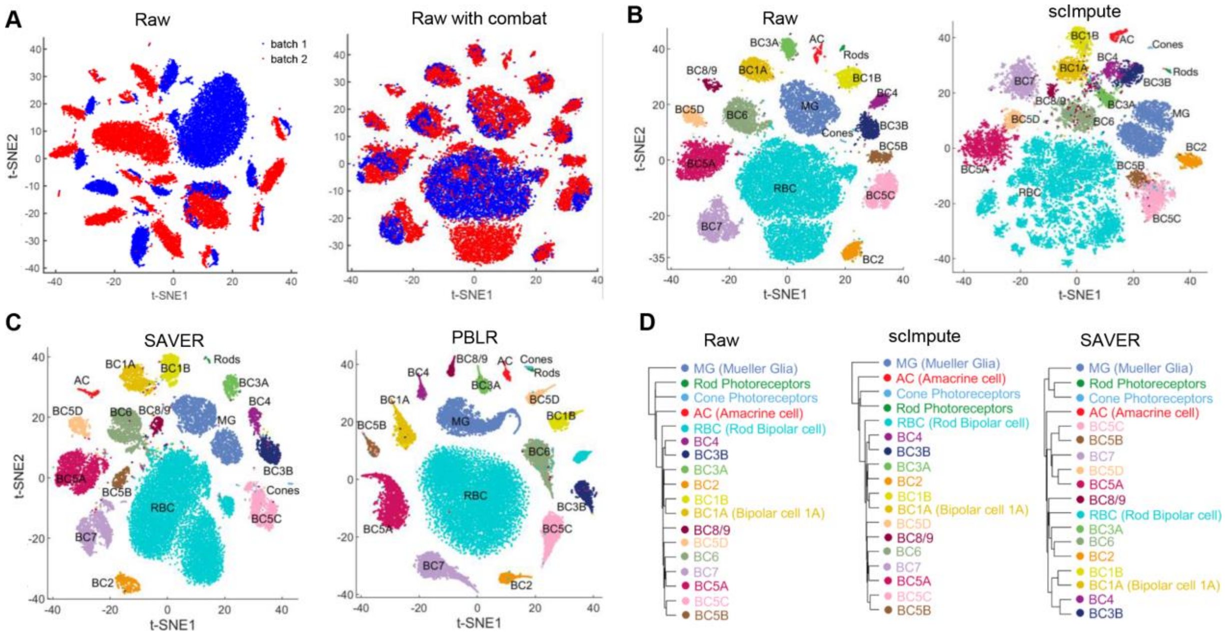

Figure S13. The imputation performance on the Shekhar dataset. (A) Cells (n = 26830) are visualized by the first two t-SNE components of the raw data and batch corrected raw data by combat. **(B)** Cells are visualized by the first two t-SNE components on raw Shekhar data and the imputed data by scImpute. **(C)** Cells are visualized by the first two t-SNE components on the imputed data by SAVER and PBLR. The initial cell groups are identified by Seurat. And batch effects are removed on these data. **(D)** Hierarchical clustering of average gene signatures of clusters based on gene expression of raw data and imputed data by scImpute and SAVER respectively (Pearson correlation distance metric, average linkage).

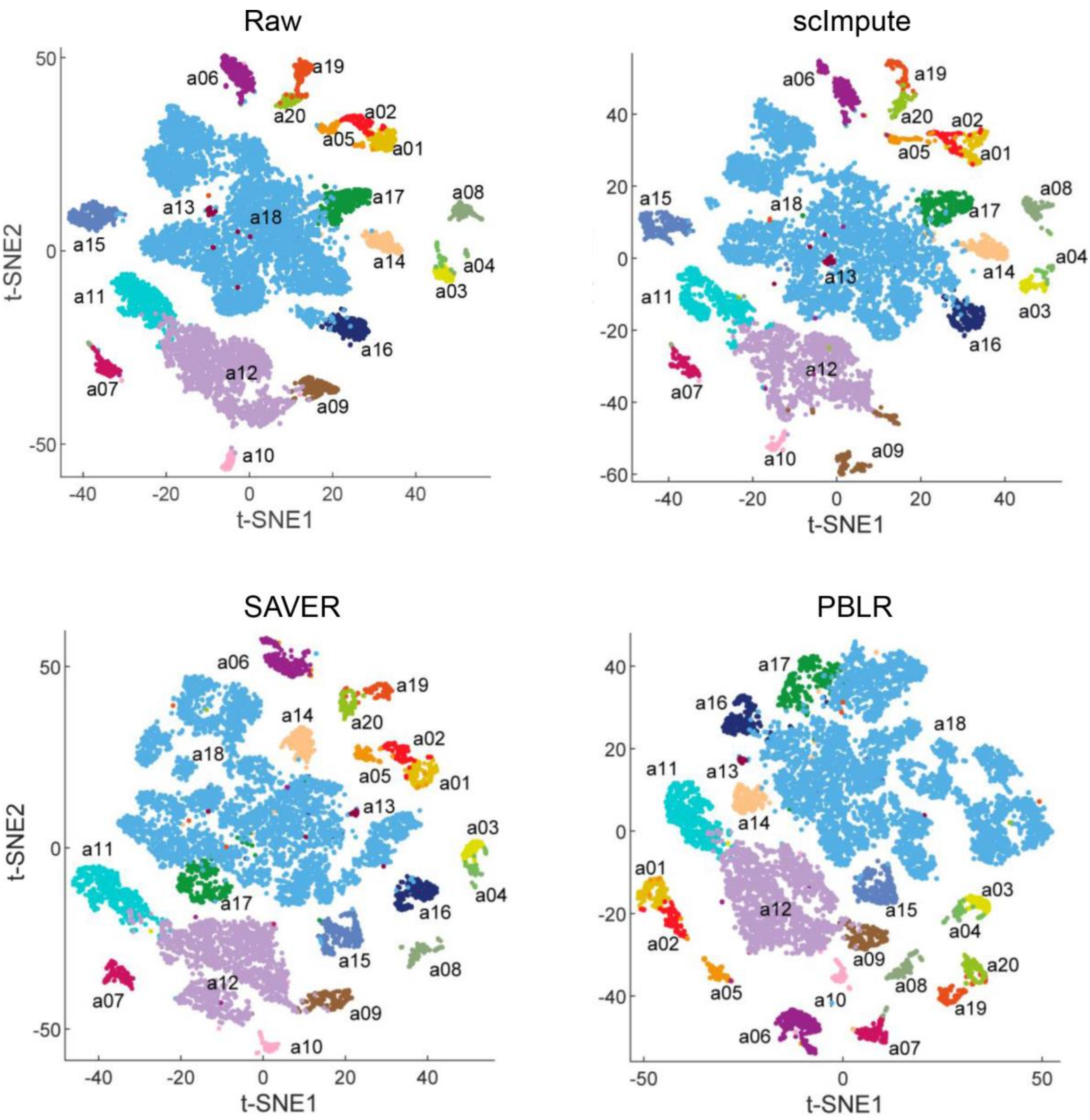

Figure S14. The imputation performance on the Campbell dataset. Downsampled cells (n = 10794) are visualized by the first two t-SNE components on the raw and imputed Shekhar dataset by scImpute, SAVER and PBLR.

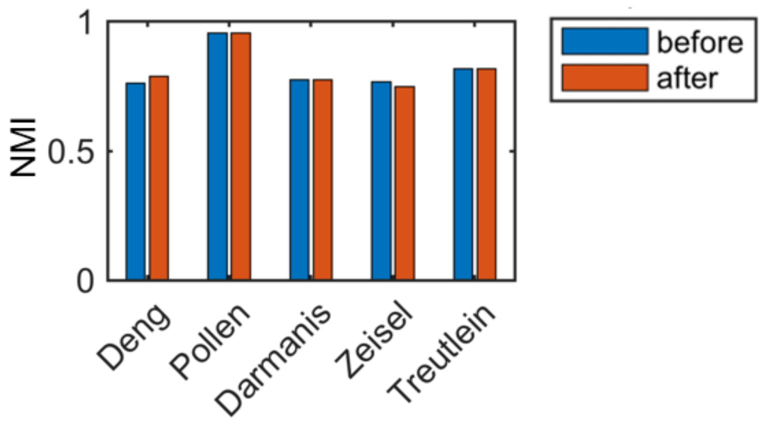

Figure S15. Comparison of cell cluster performance evaluated by NMI between before and after imputed by PBLR on five real datasets.

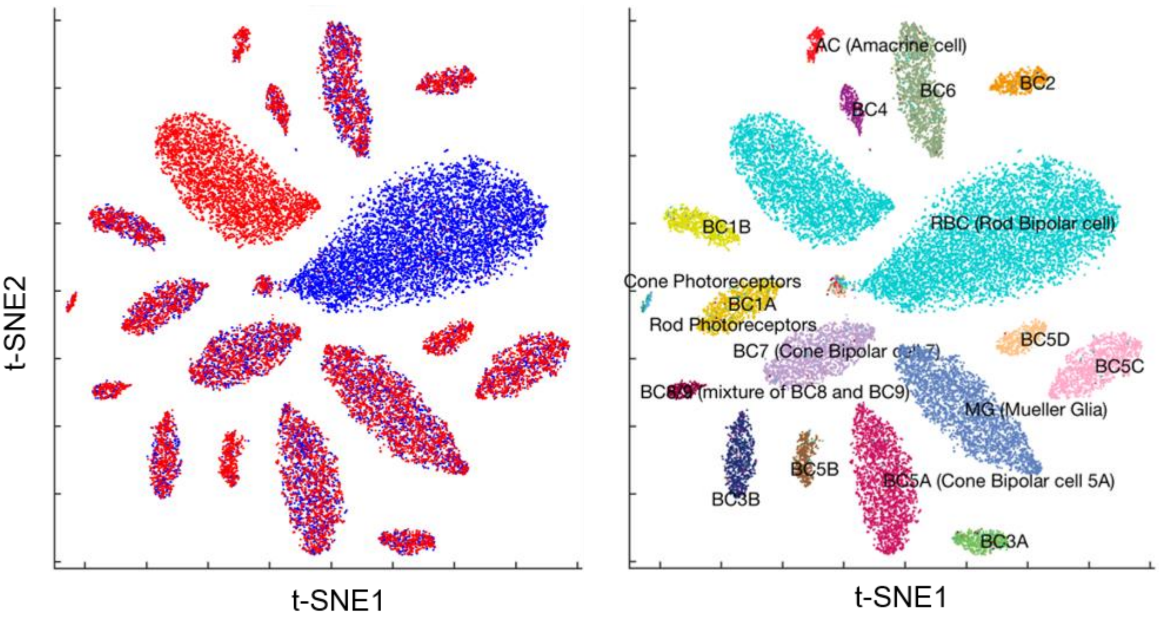

Figure S16. Cells are visualized on the first two t-SNE components using the imputed Shekhar data by PBLR with fast version of considering cell heterogeneity. Cells are colored by batches (left) and cell types (right).

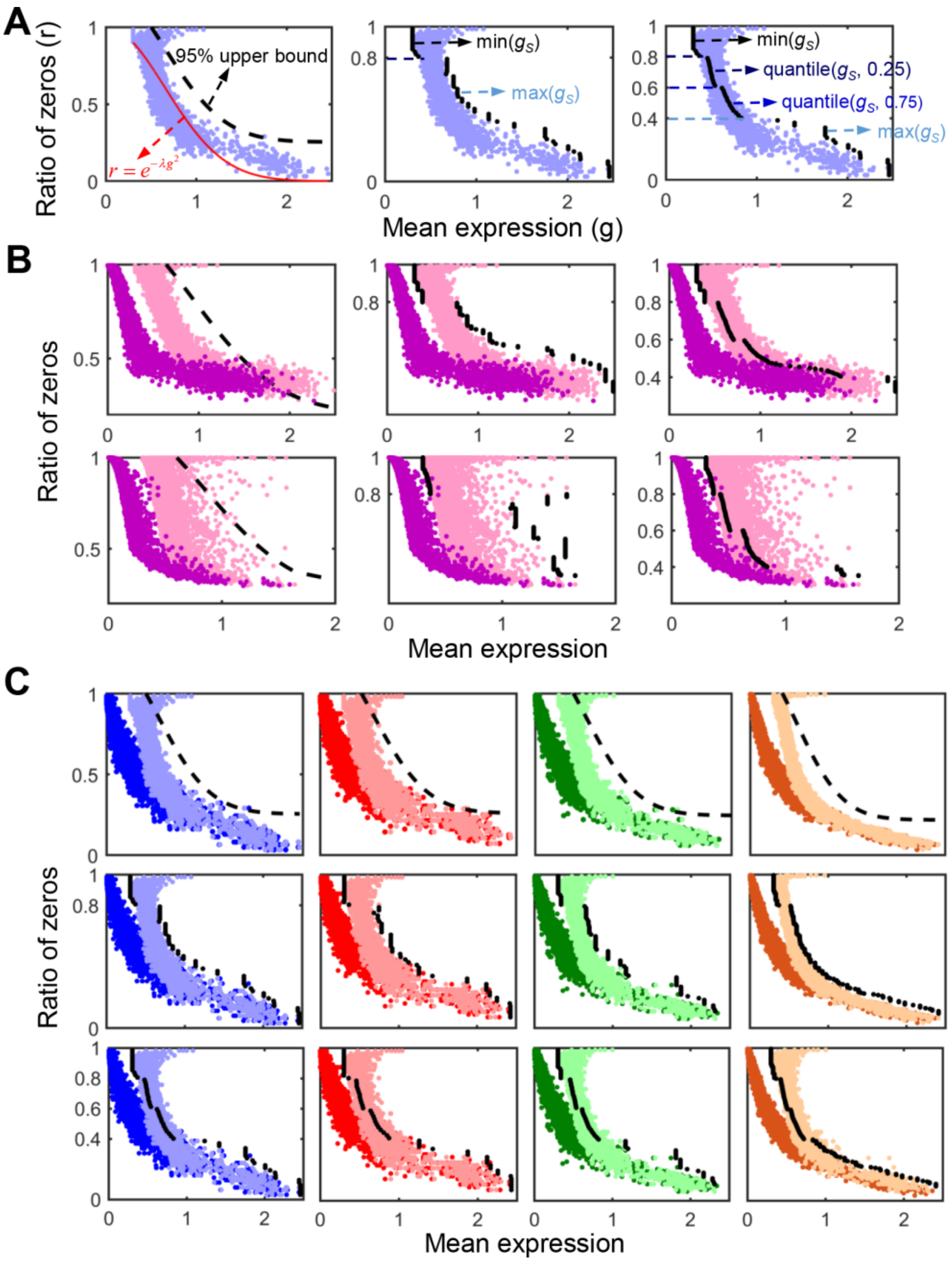

Figure S17. **Illustration of the three ways for boundary estimation. (A)** Schematic diagrams of the three ways for boundary estimation. **Left:** The boundary of each gene is defined as the upper one-sided 95% confidence bound by fitting the log-transformed mean gene expression value and the ratio of zeros using $e^{-\lambda x^2}$; **Middle:** Boundary estimation by a simple piecewise function with two sub-functions; **Right:** Boundary estimation by a sophisticated piecewise functions with four sub-functions. Scatter plots of each gene with x axis representing log-transformed mean gene expression value and y axis representing the ratio of zeros across cells. Dark color represents real values in the zero space, while light color represents values in the non-zero space. The black dots represent the estimated boundary. **(B)** Comparison of the estimated boundary based on the sampled reference data from both synthetic dataset and real dataset. **Upper:** The reference data was generated from synthetic dataset 1; **Bottom:** The reference data was generated from the real Zeisel dataset. **(C)** Scatter plots of each gene with x axis representing log-transformed mean gene expression value and y axis representing the ratio of zeros across cells of each group in synthetic dataset 1. Boundary is estimated by the exponential function (upper), the simple piecewise function (middle) and the sophisticated piecewise function (bottom), respectively. Again, we observed that the sophisticated piecewise function can accurately estimate the boundary of "dropout values" in each cell group.

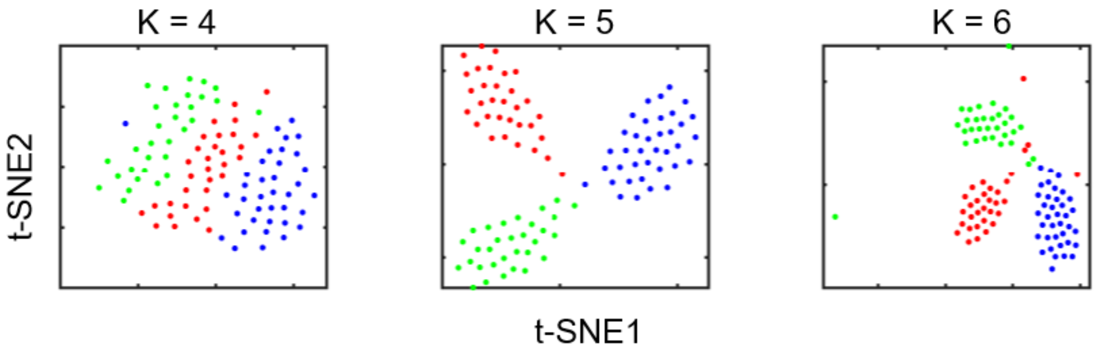

Figure S18. Robust analysis of cluster numbers. Visualization of cells on the first two t-SNE components using imputed data by PBLR with the number of clusters equaling 4, 5 and 6 respectively.

Supplementary Tables

Table S1. The parameters of Splatter used for generating synthetic datasets.

Parameter	dataset 1	dataset 2	dataset 3	dataset 4	dataset 5	dataset 6	dataset 7	dataset 8
**version**	1.4.0	1.4.0	1.2.1	1.0.1	1.0.1	1.10.1	1.10.1	1.10.1
**nGenes**	10000	1000	10000	1000	1000	1000	1000	1000
**nCells**	200	100	100	1000	100	1000	1000	1000
**group.prob**	c(0.3,0.3,0.4)	c(0.3,0.3, 0.4)	c(0.3,0.3,0.4)	c(0.24,0.12,0.1, 0.02,0.37,0.15)	Null	c(1/3,1/3,1/3)	c(1/5,1/5,1/5, 1/5)	c(0.25,0.5, 0.1,0.15)
**dropout.shape**	c(-0.5,-0.4,- 0.5)	{-0.2, -0.15, -0.1, -0.05}	Default	Default	Default	c(-0.5,-1, -1.5)	c(-0.2,-0.4, -0.6,-0.8,-1)	c(-0.5,-1, -1.5,-2)
**dropout.mid**	c(0,0,0)	Default	Default	Default	Default	c(0,0,0)	c(0,0,0,0,0)	c(0,0,0,0)
**dropout.type**	"group"	”group”	Null	Null	Null	"group"	"group"	"group"
**Method**	"groups"	"groups"	"groups"	"groups"	"path"	"path"	"path"	"groups"
**de.prob**	Default	c(0.05,0.08, 0.1)	Default	Default	Default	Default	Default	Default
**de.facLoc**	Default	0.5	Default	0.1	Default	Default	Default	{4,3,2,1}
**de.facScale**	Default	0.8	Default	0.4	Default	Default	Default	Default
**bcv.common**	Default	Default	Default	Default	Default	Default	Default	{0.1,0.2,0.3, 0.4}
**path.from**	Null	Null	Null	Null	Default	c(0,1,1)	c(0,1,1,2,2)	Null
**dropout.present**	Null	Null	TRUE	TRUE	TRUE	Null	Null	Null

Table S2. Genes enriched in TE, EPI and PE on HEE scRNA-seq dataset.

	Genes
TE	*CDX2*
EPI	*SOX2, KLF4, FOXD3, GDF3, CLDN19, NANOG*
PE	*FGFR4, TDGF1, KRT8, KRT19, CLDN3, IFTM1, DPPA5, JMJD4, NODAL*

Table S3. Marker genes of 18 cell subpopulations in Shekhar scRNA-seq dataset. These markers were obtained from the table S2 in the the original study (Shekhar et.al, 2016).

	Marker genes
RBC (Rod Bipolar cell)	Vsx2, Otx2, Grm6, Isl1, Prkca, Car8, Sebox
Müller Glia (MG)	Vsx2, Apoe, Glul, Aqp4
BC5A (Bipolar cell 5A)	Vsx2, Otx2, Scng, Grm6, Isl1, Cabp5, Hcn1, Kcng4
BC7	Vsx2, Otx2, Grm6, Isl1, Vsx1
BC6	Vsx2, Otx2, Scgn, Grm6, Isl1, Vsx1, Syt2
BC5C	Vsx2, Otx2, Scgn, Grm6, Isl1
BC1A	Vsx2, Otx2, Scgn, Tacr3
BC3B	Vsx2, Otx2, Scgn, Grik1, Prkar2b
BC1B	Vsx2, Otx2, Scgn, Tacr3
BC2	Vsx2, Otx2, Scgn, Tacr3, Syt2, Rcvrn
BC5D	Vsx2, Otx2, Isl1, Cabp5, Hcn1, Kcng4
BC3A	Vsx2, Otx2, Scgn, Irx6, Hcn4
BC5B	Vsx2, Otx2, Scgn, Grm6, Isl1, Cabp5, Hcn1
BC4	Vsx2, Otx2, Scgn, Grik1
BC8/9	Vsx2, Otx2, Grm6, Isl1
Amacrine cells (AC)	Pax6, Tfap2a, Gad1, Slc6a9
Rod photoreceptors	Rho, Pdc, Nrl, Pde6a
Cone photoreceptors	Arr3, Opn1mw, Opn1sw, Pde6h

Table S4. Marker genes of 20 cell subpopulations in Campbell scRNA-seq dataset. These marker genes were obtained from the figure 1d in the original study (Campbell et.al, 2017)**.**

	Marker genes
a01.Oligodend3	Gm21984
a02.Oligodend2	Mag, Man1a
a03.Endothelial Cells	Slco1c1
a04.Mural Cells	Mustn1
a05.Oligodend1	Bmp4
a06.NG2/OPC	Cspg4
a07.PVMMicro	Aif1
a08.VLMC	Col1a1, Col3a1, Lum
a09.Ependymocytes	Ccdc153
a10.Astrocyte	Gfap
a11.Tanycyte1	Adm
a12.Tanycyte2	Crym
a13.Neurons1	Oxt
a14.Neurons2	Rgs16
a15.Neurons3	Tac2
a16.Neurons4	Ghrh
a17.Neurons5	Slc18a2
a18.Neurons6	Tubb3
a19.ParsTuber1	Cyp2f2
a20.ParsTuber2	Tshb, Timeless

Table S5. Description of five real datasets used in this study for cell subpopulation identification.

Dataset	#genes vs #cells	Ratio of zeros (%)	#clusters
Deng	22431 vs 268	60.5	6
Pollen	23730 vs 301	67.1	11
Darmanis	20214 vs 90	80.8	9
Zeisel	19972 vs 3005	81.2	9
Treutlein	23271 vs 80	90.2	5
